# Pemphigus Vulgaris Autoantibody Profiling by Proteomic Technique

**DOI:** 10.1371/journal.pone.0057587

**Published:** 2013-03-07

**Authors:** Mina Kalantari-Dehaghi, Grant J. Anhalt, Michael J. Camilleri, Alex I. Chernyavsky, Sookhee Chun, Philip L. Felgner, Algis Jasinskas, Kristin M. Leiferman, Li Liang, Steve Marchenko, Rie Nakajima-Sasaki, Mark R. Pittelkow, John J. Zone, Sergei A. Grando

**Affiliations:** 1 Department of Dermatology, University of California Irvine, Irvine, California, United States of America; 2 Department of Dermatology, Johns Hopkins University, Baltimore, Maryland, United States of America; 3 Department of Dermatology, Mayo Clinic, Rochester, Minnesota, United States of America; 4 Department of Medicine, University of California Irvine, Irvine, California, United States of America; 5 Department of Dermatology, University of Utah, Salt Lake City, Utah, United States of America; 6 Department of Biological Chemistry, University of California Irvine, Irvine, California, United States of America; 7 Institute for Immunology, University of California Irvine, Irvine, California, United States of America; University of Tennessee, United States of America

## Abstract

Pemphigus vulgaris (**PV**) is a mucocutaneous blistering disease characterized by IgG autoantibodies against the stratified squamous epithelium. Current understanding of PV pathophysiology does not explain the mechanism of acantholysis in patients lacking desmoglein antibodies, which justifies a search for novel targets of pemphigus autoimmunity. We tested 264 pemphigus and 138 normal control sera on the multiplexed protein array platform containing 701 human genes encompassing many known keratinocyte cell-surface molecules and members of protein families targeted by organ-non-specific PV antibodies. The top 10 antigens recognized by the majority of test patients’ sera were proteins encoded by the DSC1, DSC3, ATP2C1, PKP3, CHRM3, COL21A1, ANXA8L1, CD88 and CHRNE genes. The most common combinations of target antigens included at least one of the adhesion molecules DSC1, DSC3 or PKP3 and/or the acetylcholine receptor CHRM3 or CHRNE with or without the MHC class II antigen DRA. To identify the PV antibodies most specific to the disease process, we sorted the data based on the ratio of patient to control frequencies of antigen recognition. The frequency of antigen recognition by patients that exceeded that of control by 10 and more times were the molecules encoded by the CD33, GP1BA, CHRND, SLC36A4, CD1B, CD32, CDH8, CDH9, PMP22 and HLA-E genes as well as mitochondrial proteins encoded by the NDUFS1, CYB5B, SOD2, PDHA1 and FH genes. The highest specificity to PV showed combinations of autoantibodies to the calcium pump encoded by ATP2C1 with C5a receptor plus DSC1 or DSC3 or HLA-DRA. The results identified new targets of pemphigus autoimmunity. Novel autoantibody signatures may help explain individual variations in disease severity and treatment response, and serve as sensitive and specific biomarkers for new diagnostic assays in PV patients.

## Introduction

Pemphigus vulgaris (**PV**) is a mucocutaneous blistering disease characterized by IgG autoantibodies against stratified squamous epithelium. PV antibodies demonstrate epithelial cell-surface staining by indirect immunofluorescence (**IIF**), and, because this staining appears between cells, initially the antibodies were described as “intercellular” antibodies [1], [2]. Although the incidence of PV is only 1 to 16 per million population per year [3], [4], this disease represents a significant burden to health care professionals, and the health care system. Systemic administration of glucocorticosteroid hormones is essential to establish control of disease during the acute stage [5]. While glucocorticosteroid treatment is life saving, it may cause severe side effects, including death [6], [7]. The development of non-steroidal treatment has been hampered by a lack of clear understanding of the mechanisms leading to keratinocyte detachment in PV.

During the last decade, the studies of autoimmune responses in PV have been supplemented and, to some extent, replaced by analyzing the levels of antibodies to desmoglein (**Dsg**) 3 by enzyme linked immunosorbent assay (**ELISA**) representing a hallmark and a diagnostic criterion of PV [8]. However, Dsg 3 antibody levels do not always correspond to the presence of cell-surface antibodies by IIF or correlate with disease activity [9], [10], [11] or predict relapse of the disease [12]. Furthermore, anti-Dsg antibodies can be absent in the active stage of disease but present in PV patients during remission [13], [14], [15], [16], [17], [18], patients with unrelated medical conditions, and healthy subjects, including relatives of PV patients [17], [19], [20], [21], [22], [23], [24], [25], [26]. For example, 16 PV patients positive for cell-surface antibodies by IIF had normal Dsg 3 antibody levels [27].

Identification of proteins targeted by autoantibodies in PV is a subject of intense research. The first evidence that keratinocyte antigens other than Dsg 1 and Dsg 3 are pathophysiologically relevant was provided by experiments showing the ability to induce suprabasal acantholysis and gross skin blisters in *Dsg3^−/−^* neonatal mice by passive transfer of PV antibodies [28]. In this model, murine epidermis lacks Dsg 3 and the passively transferred PV IgG lacks Dsg 1 antibody. Hence, the injected PV antibodies cause blisters by targeting non-Dsg 1 and Dsg 3 keratinocyte antigens. Current understanding, however, does not adequately explain the mechanism of acantholysis in patients lacking Dsg 1 and 3 antibodies. Furthermore, results of a recent study indicate that autoreactivity in PV relies on somatic mutations generated in response to an antigen unrelated to Dsg 3 [29]. Taken together, these facts justify a search for novel targets of pemphigus autoimmunity.

In general, autoimmune diseases are characterized by the presence of multiple types of autoantibodies mediating a coordinated immunological attack against a fraction of the tissue proteome. For example, 116 autoantibodies were described in patients with systemic lupus erythematous [30]. The number of targeted self- antigens varies dramatically from patient to patient. Therefore, multiplex analysis of autoantibody responses against a spectrum of candidate antigens represents a powerful screening tool to delineate biomarker signatures in autoimmunity, allowing elucidation of the overall autoimmune process rather than individual components [31]. The availability of multiplex technologies has made possible the simultaneous detection of several different autoantibodies overcoming some of the limitations of conventional methods [32]. For instance, antigen arrays proved to be 4- to 8-fold more sensitive than conventional ELISA analyses for detection of autoantibodies specific for some autoantigens [33]. Thus, autoantibody profiling may serve purposes including classification of individual patients and subsets of patients based on their “autoantibody fingerprint,” examination of epitope spreading and antibody isotype usage, discovery and characterization of candidate autoantigens, and tailoring antigen-specific therapy [34], [35].

Historically, studies of autoimmune responses had been conducted by analyzing the presence and/or concentration of single antibodies in biological fluids using conventional immunoassays, such as ELISA, radioimmunoassay, immunoblot, and others. More recently, antigen microarrays have been constructed and validated for over a dozen autoimmune diseases, including connective-tissue diseases, primary biliary cirrhosis, experimental autoimmune encephalomyelitis, multiple sclerosis, rheumatoid arthritis, diabetes, Crohn’s disease and sclerosing cholangitis [36]. Previous reports demonstrated feasibility of the protein microarray as a laboratory tool allowing parallel analysis against hundreds of different antigens in minimal serum quantities (less than 2 µL). We pioneered the use of multiplexed protein array platforms to evaluate PV autoantibody profiles [37]. In our previous study, the sera from acute PV patients and healthy donors were probed using the microarray containing self-antigens characteristic of the organ-non-specific autoimmune disorders, such as rheumatoid arthritis, lupus erythematosus, scleroderma, diabetes and some other autoimmune disorders [37]. The results identified the presence of several non-organ specific antibodies, but the relatively small sample size did not allow determination of their prevalence in PV patients. Most recently, some of these new autoantibodies were validated in an independent proteomic study [38], indicating reliability of identifying novel disease-specific autoantibodies in PV through multiplexed parallel testing.

To elucidate the immunopathological mechanisms underlying keratinocyte detachment in PV, we designed a multiplexed protein array platform encompassing most of known keratinocyte cell-surface molecules as well as members of protein families targeted by organ-non-specific PV antibodies. In the present study, we utilized this specialized microarray to test a large number of PV patient and control sera to define the proteome targeted by PV autoimmunity. As predicted by the multiple hit hypothesis [39], an in-depth analysis of PV sera revealed new self-antigens and identified specific patterns of differentially reacting autoantibodies. Such novel autoantibody signatures may help explain individual variations of disease severity and response to treatment in PV patients, and serve as sensitive and specific biomarkers for new diagnostic assays.

## Materials and Methods

### Test Sera

We tested 264 PV patient and 138 normal serum specimens. Patient specimens were selected based on IIF results showing the presence of cell-surface antibodies staining stratified squamous epithelial substrate (human skin and/or monkey esophagus purchased from California National Primate Research Center, Davis, CA; http://www.cnprc.ucdavis.edu/) in the characteristic pemphigus pattern at a serum titer of 1∶40 concentration and higher. The Dsg3 antibody level was measured using the MESACUP Dsg3 ELISA test system (MBL International Corp., Nagoya, Japan). Patient specimens were de-identified prior to testing. As controls, we used sera collected from healthy donors at University of California Irvine and normal human sera purchased from Bioreclamation, Inc. (Westbury, NY). This research has been approved by Institutional Review Boards (IRB) at University of California Irvine. Participants provided their written informed consent on the IRB-apprroved consent forms to participate in this study.

### Microarray Design and Printing

**Figure 1:**
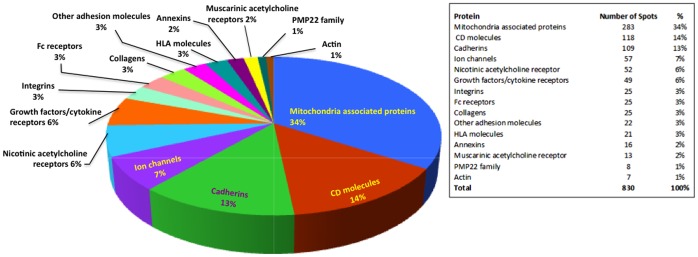
Composition of the multiplexed protein array platform.

Protein microarray construction consisted of four-steps: (i) PCR amplification of each complete or partial open reading frame (**ORF**); (ii) *in vivo* recombination cloning; (iii) *in vitro* transcription/translation; and (iv) microarray chip printing. ORFs were obtained from the National Institutes of Health Mammalian Gene Collection clones (Invitrogen, Carlsbad, CA and MCLAB, San Francisco, CA), and amplified using custom polymerase chain reaction (**PCR**) primers comprising 20 base pairs of gene-specific sequences with 15 base pairs of “adapter” sequences. The size of amplified genes ranged from 246 to 6786 base pair. ORFs >4,000 base pairs were cloned as 3 overlapping segments. The adapter sequences, which became incorporated into the termini flanking the amplified gene, were homologous to the cloning site of the linearized T7 expression vector pXT7 allowing the PCR products to be cloned by *in vivo* homologous recombination in competent DH5α cells [40], [41]. The resulting fusion protein also incorporated a 5′ polyhistidine epitope, an ATG translation start codon, and a 3′ hemagglutinin epitope and T7 terminator. Plasmids were expressed in 5 h *in vitro* transcription/translation reactions (rapid translation system, **RTS,** 100 *E. coli* HY kits; Roche, Switzerland) according to the manufacturer’s instructions. Protein expression was monitored by microarray using monoclonal anti-polyhistidine (clone His-1; Sigma Chemical Co., St Louis, MO) and anti-hemagglutinin (clone 3F10; Roche). Microarrays were printed onto Whatman nitrocellulose coated glass FAST slides using an Omni Grid 100 microarray printer (Genomic Solutions, UK). Each microarray chip consisted of proteins representing 701 unique human genes and their transcript variants, and peptides of the Dsg 1 and Dsg 3 extracellular domains 1–5 (**Table S1;** **Fig. 1**). The microarrays also included the following controls: (i) the “no DNA” negative control, in which an empty plasmid vector was placed in the RTS reaction; (ii) serially diluted human IgG (a positive control); and (iii) serially diluted EBNA-1 (another positive control, given the high prevalence of latent Epstein Barr viral infection).

### Antibody Assays and Reading of Microarrays

Prior to array staining, experimental and control serum samples were diluted to 1/25 in Whatman’s Protein Array Blocking Buffer containing *Escherichia coli* lysate at a final concentration of 30% and incubated at room temperature for 1 h with constant mixing. The arrays were rehydrated in the blocking buffer for 30 min that was replaced with preabsorbed test sera and incubated overnight at 4°C with constant agitation. The slides were washed five times in 10 mM Tris (pH 8.0)-150 mM NaCl containing 0.05% Tween 20 buffer, and bound antibodies were detected after 1 h incubation in Biotin-SP conjugated affinity purified human goat secondary antibody to IgG Fc (Fc-γ fragment specific) (Jackson Immunoresearch, West Grove, PA) diluted 1/200 in blocking buffer. The slides were washed three times, and bound antibodies detected by 1 h incubation with StreptAvidin conjugated with the dye PBXL-3 as tertiary antibody (streptavidin-conjugated SureLight® P-3) (Columbia Biosciences Corporation, Columbia, MD) diluted 1/200 in blocking buffer. After being washed three times, the slides were air-dried under brief centrifugation and stored at 18°C in a desiccator. The arrays were examined with a Perkin Elmer ScanArray Express HT apparatus at a wavelength of 670 nm and intensities were quantified using ProScanArray Express software (Perkin Elmer, Waltham, MA). All signal intensities were corrected for spot-specific background.

### Data Analysis

Signal intensity was obtained from ScanArray and background signal was corrected by subtracting “no DNA” negative control plus 2 standard deviations (**SD**) from the signals in each antigen. This standardized signal was used to determine positives in response to antigen on array. The positives were defined as the signal intensity higher than the average signal intensity of control group plus 2 SD. A two-tailed unpaired Student’s t-test was used to verify the significance of differences between the groups, and *p* values less than 0.05 were considered significant. The microarray’s ability to detect disease-related antibodies was evaluated based on the ratio of positive patient *vs.* control sera. JMP Software (SAS Institute Inc., Cary, NC) was used to perform the principal component analysis as detailed elsewhere [42].

## Results

### Self-antigens Targeted by Pemphigus Antibodies

**Figure 2:**
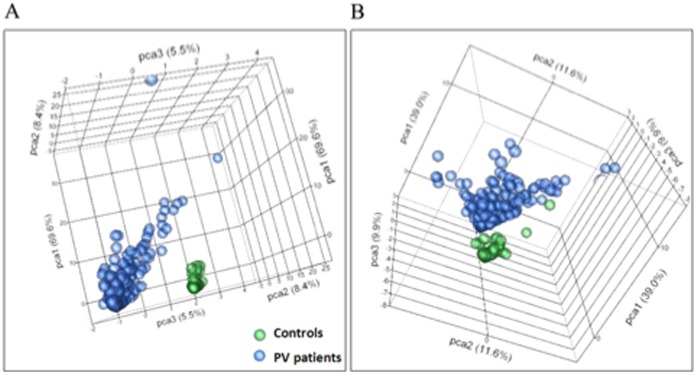
Principal component analysis of top antigens. Unsupervised principal component analysis of the signal intensity for samples from PV patients and healthy controls revealed that these two groups could be segregated on the basis of top 30 antigens with the highest sensitivity (**A**) and 15 antigens with the highest specificity (**B**) for PV.

Analyses of the data revealed positive reactions of both PV patient and control sera with a large number of peptides included in the microarray (**Table S1**). The specificity was determined by the frequency of antigen recognition by PV sera. To identify the PV antibodies most specific to the disease process, we re-sorted the data based on the ratio of patient to control frequencies of antigen recognition in the microarray. The unsupervised principal component analysis demonstrated that the top 30 antigens recognized by PV antibodies with the highest sensitivity or top 15 antigens recognized with the highest specificity were clearly distinct from controls (**Fig. 2**). These results indicated that PV features a unique serological profile that distinguishes pemphigus autoimmunity from normal immune response.

**Figure 3:**
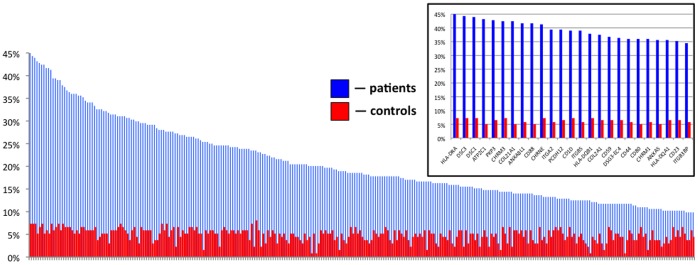
Sensitivity of the microarray in detecting disease-related autoantibodies. Sorted by percent of positive samples in the group of antigens recognized by 10 percent and more of PV patients. ***Inset:*** 25 top antigens.

The sensitivity analysis (**Fig. 3**) demonstrated that the majority of patients’ sera targeted the DR α chain of the class II major histocompatibility complex (**MHC**) encoded by the human leukocyte antigen (**HLA**)-DRA gene (45% PV patients), desmocollin 1 and desmocollin 3 (DSC1 and DSC3, respectively; 44% each), ATPase, Ca^++^ transporting, type 2C, member 1 (ATP2C1; 43%), plakophilin 3 (PKP3; 43%), M_3_ subtype of muscarinic acetylcholine receptor (**AChR**) (CHRM3; 42%), collagen α1, type XXI, (COL21A1; 42%), annexin A8-like 1 molecule (ANXA8L1; 42%), complement component 5a receptor 1 (CD88; 42%) and ε subunit of nicotinic AChR (CHRNE; 41%).

**Figure 4:**
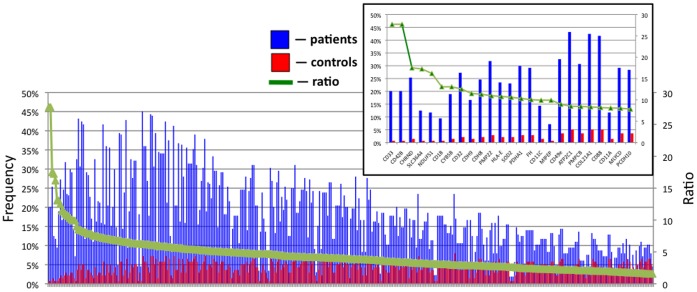
Specificity of the microarray in revealing disease-specific PV antibodies. Sorted by ratio of positive patient/control samples in the group of antigens recognized by two and more time frequently by PV patient than control sera. ***Inset:*** 25 top antigens.

The specificity analysis (**Fig. 4**) demonstrated that self-antigens recognized by PV antibodies with the frequency that exceeded that of control by 10 and more times included the cell-surface molecules sialic acid-binding immunoglobulin-like lectin 3 (CD33; ratio  = 27.7) and glycoprotein Ibα (GP1BA; 27.7), δ subunit of nicotinic AChR (CHRND; 17.6), proton-coupled amino acid transporter 4 (SLC36A4; 17.3), the antigen-presenting protein CD1B (13.1), Fc-fragment of IgG (CD32; 12.5), cadherins 8 (CDH8; 11.3) and 9 (CDH9; 11.5), peripheral myelin protein 22 (PMP22; 11.0), the MHC class I molecule E (HLA-E; 10.8) and the mitochondrial proteins NADH-ubiquinone oxidoreductase (NDUFS1; 16.2), cytochrome b5 outer mitochondrial membrane isoform precursor (CYB5B; 13.1), superoxide dismutase (SOD2; 10.6), α subunit of pyruvate dehydrogenase E1 component (PDHA1; 10.3) and fumarate hydratase (FH; 10.1).

### Non-Dsg 3 Antibodies Produced by the Dsg 3 Antibody-positive PV Patients

Although sera from all 264 patients demonstrated a pemphigus cell-surface staining pattern by IIF, only 183 (69%) of them recognized at least one Dsg 3 peptide in the microarray (**Table S1**). This was not surprising, because Amagai’s group reported that only 60 to 75 percent of PV patients develop autoantibodies recognizing recombinant Dsg 3 proteins produced in a bacterial expression system [43]. In our study, the Dsg 3 antibody-negative patient sera likely were from PV patients who did not have detectable levels of Dsg 3 antibody, which is not uncommon [16], and/or pemphigus foliaceus (**PF**) patients, since both PV and PF antibodies demonstrate an indistinguishable IIF staining pattern [21], [44], [45]. Therefore, to extrapolate our results specifically to the immunopathology of PV, we separately determined antigen reaction patterns of the Dsg 3 antibody-containing patient sera (**Table S2**). The antigen reactivity pattern of the Dsg 3 antibody-positive sera was similar to that of the entire collection of patients’ sera (**Table S1**). Most importantly, the top 10 most frequently recognized self-antigens were identical, but displayed a slightly different order within the group (**Table 1**). Therefore, the microarray results of the entire serum collection were representative of PV autoimmunity.

**Table 1:** Top 10 antigens most frequently recognized by PV antibodies.

Symbol	Name	% of all patient samples	% of Dsg3-antibody positive samples
HLA-DRA	major histocompatibility complex, class II, DR α chain	45	53
DSC3	desmocollin 3 isoform Dsc3a preproprotein	44	54
DSC1	desmocollin 1 isoform Dsc1a preproprotein	44	54
ATP2C1	ATPase, Ca^++^ transporting, type 2C, member 1	43	50
PKP3	plakophilin 3	43	53
CHRM3	cholinergic muscarinic receptor type 3	42	51
COL21A1	collagen, type XXI, α1	42	50
ANXA8L1	annexin A8-like 1	42	49
CD88	complement component 5a receptor 1	42	50
CHRNE	nicotinic acetylcholine receptor ε subunit	41	50

### Combinations of most Common Individual Antigens Targeted by Antibodies in PV Patients

**Figure 5:**
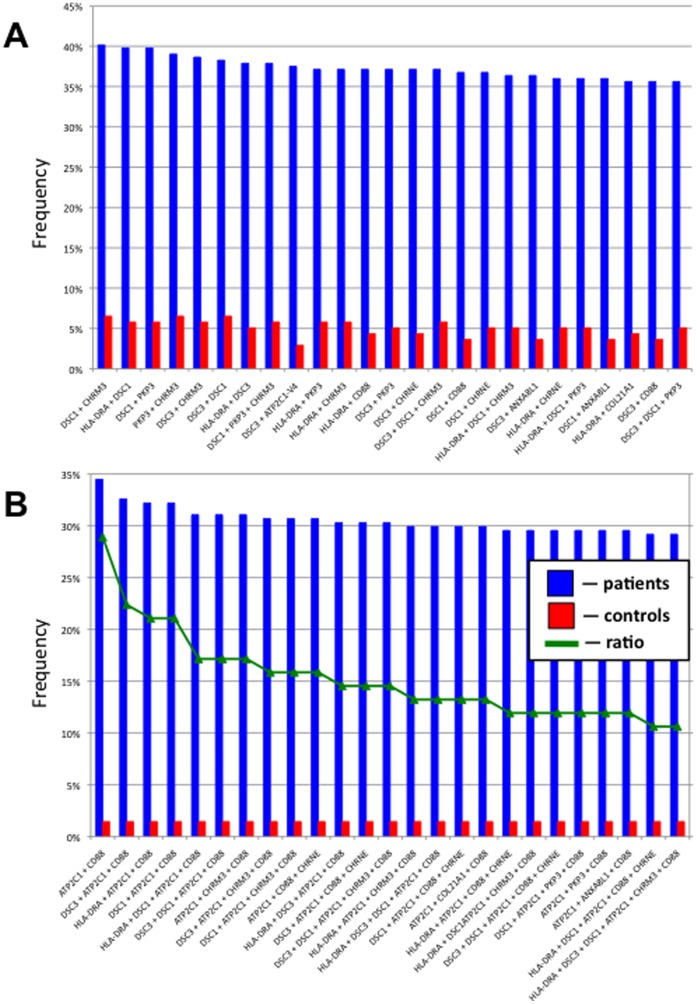
Combinations of top 10 most common individual antigens targeted by PV antibodies. **A,** Top 25 combinations sorted by percent of positive samples. **B,** Top 25 antigen sorted by the patient/control ratio.

The top 10 most commonly recognized antigens (**Table 1**) were subjected to an association analysis. We combined two and more from the top 10 most commonly recognized antigens and calculated the number of patient and control sera that contained antibodies against those combined antigens, then computed the ratio of patient over control sera positivity. The most common combinations of target antigens included at least one of the adhesion molecules DSC1, DSC3 and PKP3 and/or the AChR CHRM3 or CHRNE with or without the MHC class II antigen DRA (**Fig. 5A**). The highest specificity to PV showed combinations of antibodies to the calcium pump encoded by ATP2C1 with CD88 (C5a receptor) plus DSC1 or DSC3 or HLA-DRA (**Fig. 5B**).

## Discussion

In this study, we used sera from a large cohort of pemphigus patients to identify novel targets for PV antibodies. We applied a protein microarray approach to characterize the autoantibody response profile associated with the disease process. This approach has been employed broadly to identify differentially reactive, serodiagnostic antigens against infectious agents, and is now also being used in autoimmune diseases. In the past, our group has extensively characterized disease-associated antibody profiles in large cohorts of healthy and infected individuals (e.g., [42], [46]), and also initiated the proteomic analysis of pemphigus autoimmunity [37]. The pilot study of PV sera demonstrated that the reactivities of novel PV antibodies correlate closely with the Dsg 3 antibody levels [37]. In the present study, we used polypeptides representing different regions of Dsg 3 because, at variance with early studies [47], [48], it has been recently demonstrated that the Dsg 3 autoantibody response in PV is polyclonal [29]. We also tested autoantibody reactivities with other proteins implicated in PV pathogenesis, such as AChRs [39].

We sought to reconcile the knowledge about important role of Dsg 3 antibody in PV with the evidence that Dsg 3 is not essential for keratinocyte adhesion and Dsg 3 antibody is not requisite for acantholysis (reviewed in [49]). The results revealed new targets of pemphigus autoimmunity, and demonstrated variability of the autoantibody profile among different PV patients. We identified both the most common and the most specific autoantibodies directed against members of the cell adhesion molecule and the AChR families, which are known targets in pemphigus, as well as a number of new organ-specific and non-specific targets from previously unsuspected protein families. Based on these data, it is apparent that the immunopathology of PV is complex and variable, inducing alterations in vital keratinocyte functions due to a simultaneous hits by an array of autoantibodies of different antigenic specificities. Targeting of non-Dsg molecules potentially exacerbates the pathogenic effects of Dsg 3 antibody. Our new PV autoantibody database should provide a roadmap to navigate pemphigus research toward new disease pathways and treatment approaches.

An important role of the conformational epitope of self-antigens in PV has been vividly demonstrated in the studies of the recombinant Dsg 3 species that were raised in different expression systems and had various posttranslational modifications [43], [50], [51], [52]. An inherent drawback of the high-throughput approach applied here is that not all proteins on the array likely are folded in the same way as in human cells, and/or that they are not post-translationally modified in the same way. Consequently, they may not display all possible antigenic epitopes. This potential limitation, however, did not prevent us from reaching the main objective of our study, which was identification of new pathogenic PV autoantibodies that may work together with Dsg 3 antibodies to disrupt keratinocyte adhesion. Hundreds of autoantigens were found to be significantly more reactive with PV than control sera. Importantly, the top antigenic targets significantly separated PV patients from healthy individuals, demonstrating strong sensitivity and specificity. This observation indicated that the two approaches to microarray data analysis can be used together. The proteomic approach, therefore, serves as a starting point to “rule in,” but not necessarily “rule out,” individual self-antigens or their combinations that contribute to the autoimmune response.

The results indicate that acantholysis in PV likely derives from simultaneous inactivation of several physiological pathways maintaining keratinocyte adhesion, as predicted by the “multiple hit” hypothesis of pemphigus pathophysiology [39]. In addition to Dsg 3, other keratinocyte adhesion molecules are presumably destroyed or inactivated by PV antibodies. Notably, previous results showing that chimeric proteins containing the extracellular epitope of Dsg 1 or Dsg 3 combined with the Fc portion of human IgG absorbed out all disease-causing pemphigus IgGs [50], [53], [54] should be interpreted with caution, because both this and previous [37] proteomics studies demonstrated that PV patients produce autoantibodies against Fc-IgG.

Our results are consistent with previous reports that desmocollins [55], [56] and classical cadherins [57], [58] also are targeted by pemphigus antibodies. The significance of the autoantibody targeting DSC3 in PV is underscored by the evidence that: (i) the DSC3 loss of function mouse model exhibits phenotypic similarity to PV [59], (ii) adsorption with DSC3 can eliminate acantholytic activity of PV IgG [60], and (iii) DSC3 monoclonal antibody causes intraepidermal blistering in *in vitro* model of human skin and loss of cell-cell adhesion in keratinocyte cultures [61]. Indeed, production of certain autoantibodies against cell adhesion molecules and structural proteins, such as the intracellular desmosomal plaque protein, plakophilin 3, and the collagen α1, type XXI, may be secondary to acantholysis, as discussed in detail elsewhere [62].

The high percentage of patients’ sera reacting with AChRs of the muscarinic and/or the nicotinic classes is in keeping with previous reports that downstream signaling from these receptors regulates keratinocyte cell-cell adhesion through physiological control of phosphorylation/dephosphorylation of desmosomal and classical cadherins [63]. Blocking AChRs expressed on keratinocytes leads to disassembly of desmosomal and adherence junctions due to phosphorylation of key adhesion molecules. Our early reports that PV and PF patients develop antibodies to keratinocyte AChRs [28], [64] and that cholinomimetic drugs can ameliorate pemphigus [65] have been recently corroborated by new clinical and laboratory data. AChR antibodies in PV patients correlate with disease extent at the time of diagnosis and during follow-up [66], and, in a patient with bipolar disorder, cholinolytic drugs worsen PF [67].

The high reactivity of PV sera with the annexin A8-like molecule was not surprising either, because it had been reported that PV patients develop antibodies to different annexins [68]. Probing of keratinocyte λgt11 cDNA library with the PV IgG eluted from a 75 kD band that stained epidermis in a pemphigus-like cell-surface pattern and caused acantholysis in keratinocyte monolayers revealed a novel type of AChRs, termed pemphaxin (a.k.a. annexin 9) [69]. Annexin A8 is specifically expressed in adult stratified epithelia [70], where it may participate in the organization of certain actin associated membrane domains and regulate late endosome organization and function [71]. By analogy to annexins 1, 2, 3 and 9 that act as non-professional AChRs [69], [72], the annexin A8-like molecule also may mediate acetylcholine signaling and, thus, be involved in regulation of keratinocyte shape and adhesion. This possibility needs further investigation.

The microarray results from this study demonstrate that mitochondrial antibodies are highly specific to the PV sera. The presence of antibodies against mitochondrial proteins in PV and PF patients had been reported [73], [74]. Mitochondrial antibodies are apparently pathogenic because their absorption abolishes the ability of PV IgG to cause acantholysis both *in vitro* and *in vivo* [74]. Based on the known functions of targeted proteins, the following mitochondrial pathways may be subject to dysfunction: tricarboxylic acid cycle, oxidative phosphorylation, O_2_ respiration, and production/inactivation of reactive oxygen species (**ROS**). The mitochondrial dysfunction in PV has been directly or indirectly suggested by an increase of lipid peroxidation, reflecting an increased production of ROS [75], [76], and the peroxidant-antioxidant balance, measuring oxidative stress [77], and activation of the mitochondria-dependent intrinsic apoptotoc pathway in keratinocytes exposed to PV IgG [74], [78], [79]. At this point, however, it remains unclear how PV mitochondrial antibodies enter keratinocytes. A cell-surface protein may act as a surrogate for the nominal mitochondrial antigen. For example, since annexins can relocate to the cytosol reaching mitochondria [80], mitochondrial antibodies may enter keratinocytes bound to annexins. Additionally or alternatively, instead of binding to an antigen on the plasma membrane, an antibody may be internalized in a complex with Fc receptors expressed on keratinocytes [81], [82].

Of particular interest is a discovery in PV of autoimmunity against members of the PMP (peripheral myelin protein)-22/gas3 family termed PMP-22 and PERP (p53 apoptosis effector related to PMP-22). The high specificity of PMP22 antibody demonstrated in this study confirms our previous microarray results [37]. A relatively high prevalence of autoantibody against the structurally related PERP (31% positive patients, 5% controls), a tetraspan membrane protein originally identified as an apoptosis-associated target of the p53 tumor suppressor, was not surprising either. The relevance of anti-PERP autoimmunity to the pathophysiology of PV is underscored by the fact that PERP knockout mice display a phenotypic similarity to PV [83]. *Perp^−/−^* mice die within the first week of life as a result of severe adhesion defects and blistering of the skin and oral mucosa. Further, they exhibit highly abnormal desmosomes by electron microscopy. Dissolution of desmosomes and PV-like intraepidermal split in *Perp^−/−^* mice may result from aberrant inside-out signaling along the altered cell death pathways, because PERP is involved in the extrinsic apoptotic pathway, playing a “death receptor” role [84].

An interesting and unexpected discovery from this study is the PV autoantibody targeting the human secretory pathway Ca^2+^/Mn^2+^-ATPase, or hSPCA1, encoded by the ATP2C1 gene located on chromosome 3. One copy of this gene is mutated in patients with Hailey–Hailey disease (a.k.a. familial benign chronic pemphigus) exhibiting the clinical-and-pathological features resembling very closely PV [85], [86]. Both Hailey-Hailey disease keratinocytes [87] and normal keratinocytes treated with PV antibodies [88] exhibit altered intracellular calcium metabolism that can lead to abnormal cell-cell adhesion [89].

This microarray study also revealed previously unrecognized autoantibodies to a number of proteins known to subserve various vital functions in cell types other than keratinocytes. The findings suggest that these cells contribute to the pathophysiology of PV and/or that the targeted proteins also function in keratinocytes. The highest specificities to PV demonstrated autoantibodies to the cell-surface molecules, sialic acid-binding immunoglobulin-like lectin 3 and glycoprotein Ibα, both of which are known to be involved in cell adhesion [90], [91].

The complement component 5a receptor 1 (a.k.a. C5a receptor) is a G protein-coupled transmembrane protein [92]. Although contribution of the complement system to the pathophysiology of pemphigus is controversial [93], [94], clinical evidence suggests that it plays a pathogenic role [95]. Hence, if autoantibodies against C5a receptor inhibit the complement cascade, they may play a protective role. Alternatively, complement activation by anti-C5a receptor may facilitate acantholysis.

Autoantibodies against the antigen-presenting protein CD1B, and MHC class I and II molecules may interfere with normal functioning of the skin immune system [38]. They may also directly affect keratinocyte shape and adhesion. Incubation of skin explant culture with HLA-A, -B and -C alloantibodies has been reported to produce keratinocyte detachment and structural disorganization similar to that found in cultures treated with PV antibodies [96].

Another protein targeted by disease-specific PV antibodies, proton-coupled amino acid transporter 4, coded by the gene SLC36A4, is a widely distributed member of the solute carrier family. It is a high-affinity transporter for proline and tryptophan [97]. In the past, we have reported that PV antibody targets a novel antigen similar to the taurine transporter that controls cellular size and water content [98], but we have much to learn about transporter proteins in keratinocyte biology and pemphigus pathophysiology.

We believe that a simultaneous and, perhaps, synchronized inactivation of the physiological pathways regulating and mediating keratinocyte adhesive function is required to disrupt the most important phylogenetic function of tegumental cells, such as integrity of epidermal barrier. To further explore the “polypathogenic” nature of pemphigus autoimmunity, we looked for the most common combinations of individual autoantibodies associated with Dsg 3 autoimmunity in PV. The results demonstrated that synergy may stem from functional cooperation of antibodies to distinct proteins mediating the same biologic function, such as heterophilic *trans-*interactions of desmogleins and desmocollins within the desmosome [99]. Simultaneous blockade of both desmosomal protein partners would distort cell-cell attachment in epidermis more efficiently, compared to interference with *cis-*interactions of single-type desmosomal proteins suggested by the “monopathogenic” theory of pemphigus pathophysiology [8]. Individual variations within the constellations of pathogenic antibodies targeting molecules that mediate and regulate keratinocyte adhesion likely determine the magnitude of the “multiple hit” attack required to disrupt the integrity of epidermis in a particular PV patient and explain the clinical and immunopathological variability of PV.

In conclusion, considerable progress in elucidation of the immunopathology of pemphigus can be achieved through the use of proteomic technology enabling a large-scale characterization of immune responses against self-antigens that may be involved in development and progression of the disease process in PV. Thus, autoantibody profiling using antigen arrays is well-positioned to become an anchor technology for the development of multiplex autoantibody-based biomarker assays for use in management of pemphigus patients.

## Supporting Information

Table S1 Reactivities of patient and control sera on protein micoarrays.
(PDF)

Table S2 Reactivities of Dsg3-positive patients’ sera on protein micoarrays.
(PDF)
