# Supplementary material for: Pemphigus Vulgaris Autoantibody Profiling by Proteomic Technique
Source: PLoS One. 2013 Mar 7;8(3):e57587. doi: 10.1371/journal.pone.0057587 (PMC3591405; doi:10.1371/journal.pone.0057587)
Supplement: Table S1 — Reactivities of patient and control sera on protein micoarrays. (PDF) [file pone.0057587.s001.pdf]

Suplemental Table 1. Reactivities of patient and control sera on protein micoarrays*

Symbol	Name	Patients	Controls	Ratio
**HLA-DRA**	Major histocompatibility complex, class II, DR alpha	45%	7%	6.22
**DSC3**	Desmocollin 3 isoform Dsc3a preproprotein	44%	7%	6.12
**DSC1**	Desmocollin 1 isoform Dsc1a preproprotein	44%	7%	6.06
**ATP2C1-V4**	ATPase, Ca++ transporting, type 2C	43%	5%	8.51
**PKP3**	Plakophilin 3	43%	7%	6.56
**CHRM3**	Cholinergic receptor, muscarinic 3	42%	7%	5.85
**COL21A1**	Collagen, type XXI, alpha 1	42%	5%	8.36
**ANXA8L1**	AnnexinA8 like 1	42%	6%	7.19
**CD88, C5AR1**	Complement component 5a receptor 1 (C5AR1), mRNA	42%	5%	8.21
**CHRNE**	Cholinergic receptor, nicotinic,epsilon	41%	7%	5.70
**ITGA2**	IntegrIn alpha 2 precursor	39%	6%	6.80
**PCDH12**	Protocadherin 12 precursor	39%	7%	6.04
**CD1D**	CD1d molecule	39%	7%	5.38
**ITGB5**	Integrin, beta 5	39%	6%	6.73
**HLA-DQB1**	Major histocompatibility complex, class II, DQ beta 1	38%	7%	5.23
**COL2A1**	Collagen, type II, alpha 1	38%	7%	5.75
**CD59**	CD59 molecule, complement regulatory protein	37%	7%	5.63
**DSG3-EC4**	Desmoglein 3	36%	7%	5.58
**CD44**	CD44 molecule (Indian bloodGroup)	36%	6%	6.21
**CD80**	CD80 molecule	36%	5%	7.09
**CHRM1**	Cholinergic receptor, muscarinic 1	36%	6%	6.21
**ANXA5**	Annexin 5	36%	5%	7.02
**HLA-DQA1**	Major histocompatibility complex, class II, DQ alpha 1	36%	7%	5.46
**CD23, FCER2**	Fc fragment of IgE, low affinity II, receptor for (CD23)	35%	7%	5.40
**ITGB3BP-V1**	Integrin beta 3 binding protein, isoform 1	34%	6%	5.95
**CD86**	CD86 molecule-costimulatory signal for activation of the T-cell	34%	6%	5.88
**DSC3**	Desmocollin 3 isoform Dsc3a preproprotein	34%	6%	5.88
**HLA-G**	Major histocompatibility complex, class I,G	34%	6%	5.88
**HLA-DRB1**	Major histocompatibility complex, class II, DR beta 1	33%	7%	5.11
**CD49e, ITGA5**	Integrin, alpha 5 (fibronectin receptor, alpha polypeptide)	33%	4%	8.99
**COL1A1**	Collagen, type I, alpha 1	33%	4%	7.49
**IL1RAPL2**	InterleukIn 18 receptor accessory proteIn	33%	5%	6.42
**CD26, DPP4**	Dipeptidyl-peptidase 4	32%	5%	6.35
**PC**	Pyruvate carboxylase	32%	5%	6.35
**PMP22**	Peripheral myelin protein 22	32%	3%	10.98
**CACNA1C**	Calcium channel, voltagedependent, L type,	31%	6%	5.42
**CHRNA1**	Cholinergic receptor, nicotinic, alpha 1 isoform b	31%	6%	5.42
**HLA-DPA1**	Major histocompatibility complex, class II, DP alpha 1	31%	6%	5.42
**CD56, NCAM2**	Neural cell adhesion molecule 1	31%	7%	4.76
**CDH1**	Cadherin 1, type 1 preproprotein	31%	7%	4.29
**CHRNB4**	Cholinergic receptor, nicotinic, beta 4	31%	7%	4.76
**PCDHAC2**	Protocadherin alpha subfamily C, 2 isoform 1	31%	6%	5.36
**PERP**	PerP, TP53 apoptosis effector	31%	5%	6.05
**PMPCB**	Mitochondrial processing peptidase beta subunit	31%	4%	8.47
**ACTA2**	Actin, AlphA 2, smooth muscle, AortA	30%	6%	5.23
**CHRNA5**	Cholinergic receptor, nicotinic, alpha 5	30%	7%	4.65
**OGFR**	OpioidGrowth factor receptor	30%	4%	6.88
**PDHA1**	Pyruvate dehydrogenase E1 component alpha subunit, somatic form	30%	3%	10.32
**ANXA3**	AnnexinA3	30%	7%	4.53
**CHRM4**	Cholinergic receptor, muscarinic 4	30%	6%	5.10
**DSG1-EC5**	Desmoglein 1	30%	6%	5.10
**CHRM1**	Cholinergic receptor, muscarinic 1	29%	6%	5.03
**FCRLA**	Fc receptor-like and mucin-like 1	29%	6%	5.03

Suplemental Table 1. Reactivities of patient and control sera on protein micoarrays*

**FH**	Fumarate hydratase (Fumarase)	29%	3%	10.06
**MLYCD**	Malonyl-CoA decarboxylase	29%	4%	8.05
**PCDH10**	Protocadherin 10 isoform 1 precursor	28%	4%	7.84
**CACNG6**	Voltagedependent calcium channelGamma6	28%	5%	5.53
**CRAT**	Carnitine O-acetyltransferase	28%	7%	3.87
**ITGA4**	IntegrIn alpha 4 precursor	28%	6%	4.84
**CACNG2**	Voltagedependent calcium channelGamma2	28%	7%	3.82
**CD14**	CD14 molecule	28%	4%	6.36
**CD16a, FCGR3A**	Fc fragment of IgG, low affinity IIIa, receptor (CD16a)	28%	6%	4.77
**CD48**	CD48 molecule	28%	7%	4.24
**CD32, FCGR2B**	Fc fragment of IgG, low affinity IIb, receptor (CD32)	27%	2%	12.55
**ITGA7**	Homo sapiens mRNA for ITGA7 variant protein, partial cds, clone: hk04261.	27%	7%	4.18
**CD4**	CD4 molecule	27%	4%	6.19
**CHRNA10**	Cholinergic receptor, nicotinic, alpha 10	27%	6%	4.64
**MAOB**	Amine oxidase (flavin-containing) B	27%	5%	5.30
**CDH4**	Cadherin 4, type 1 preproprotein	27%	5%	5.23
**DSG1**	Desmoglein 1	27%	7%	4.07
**PANX1**	pannexin 1	27%	7%	4.07
**PCDHB12**	Protocadherin beta 12 precursor	27%	6%	4.57
**CD46**	CD46 molecule, complement regulatory protein	26%	7%	4.01
**JUP**	Junction plakoglobin	26%	5%	5.15
**COL8A1**	Collagen, type VIII, alpha 1	26%	5%	5.08
**CHRND**	Cholinergic receptor, nicotinic, delta	25%	1%	17.51
**PRODH**	Proline oxidase	25%	6%	4.38
**SLC6A6-V1**	solute carrier family 6 (neurotransmiter)	25%	5%	5.00
**DSC2**	Desmocollin 2 isoform Dsc2a preproprotein	25%	6%	4.31
**TGFBRAP1**	TransformingGrowth factor, beta receptor	25%	6%	4.31
**CD37**	CD37 molecule	25%	5%	4.85
**CD93**	CD93 molecule	25%	5%	4.85
**CDH8**	Cadherin 8, type 2 preproprotein	25%	2%	11.33
**CHRNB1**	Cholinergic receptor, nicotinic, beta 1 subunit	25%	6%	4.25
**DSG1-EC1**	Desmoglein 1	25%	7%	3.78
**FDXR**	NADPH:adrenodoxin oxidoreductase	25%	6%	4.25
**SOD3**	superoxide dismutase 3, extracellular	25%	5%	4.85
**CD66c, CEACAM6**	Carcinoembryonic antigen-related cell adhesion molecule 6	24%	6%	4.18
**CTNNA3-V2**	Catenin, alpha 3	24%	6%	4.18
**HLA-DQB2**	Major histocompatibility complex, class II, DQ beta 2	24%	5%	4.78
**NDUFA13**	NADH dehydrogenase [ubiquinone] 1 alpha subcomplex subunit 13	24%	6%	4.18
**CD3E**	CD3e molecule, epsilon (CD3-TCR complex)	24%	5%	4.70
**CD40**	CD40 molecule, TNF receptor superfamily member 5	24%	6%	4.12
**CHRNA1-V1**	Cholinergic receptor, nicotinic, alpha 1 isoform b	24%	6%	4.12
**NMNAT2**	Nicotinamide nucleotide adenylyltransferase 2	24%	6%	4.12
**PDK4**	Pyruvate dehydrogenase [lipoamide]] kinase isozyme 4	24%	4%	6.59
**ATP2C1-V2**	ATPase, Ca++ transporting, type 2C, member 1	23%	7%	3.24
**HLA-E**	Major histocompatibility complex, class I, E	23%	2%	10.80
**ME3**	NADP-dependent malic enzyme	23%	8%	2.95
**FGR**	Gardner-Rasheed feline sarcoma viral (v-fgr) oncogene homolog	23%	6%	3.99
**SOD2**	Superoxide dismutase [Mn]	23%	2%	10.63
**ALDH4A1**	Delta-1-pyrroline-5-carboxylate dehydrogenase	23%	5%	4.48
**FRS2**	FibroblastGrowth factor receptor substrate 2-	23%	3%	7.84
**DSG3-EC3**	Desmoglein 3	22%	6%	3.86
**HLA-E**	Major histocompatibility complex, class I, E	22%	4%	5.14
**CHRNA5**	Cholinergic receptor, nicotinic, alpha 5	22%	6%	3.79
**NDUFS6**	NADH-ubiquinone oxidoreductase 13 kDa-A subunit	22%	5%	4.33

Suplemental Table 1. Reactivities of patient and control sera on protein micoarrays*

**CD63**	CD63 molecule	22%	3%	7.45
**CHRNA2**	Cholinergic receptor, nicotinic, alpha 2	22%	5%	4.26
**FLT3**	Fms-related tyrosine kinase 3	22%	4%	4.97
**CD79**	CD79a molecule, immunoglobulin-associated alpha	21%	5%	4.18
**CHRNB4-V1**	Cholinergic receptor, nicotinic, beta 4	21%	4%	5.85
**ETFB-V2**	Electron transfer flavoprotein beta-subunit (Beta-ETF), isoform 1	21%	3%	7.32
**CD64A, FCGR1A**	Fc fragment of IgG, high affinity Ia, receptor (CD64)	20%	5%	4.03
**CDH3**	Cadherin 3, type 1 preproprotein	20%	5%	4.03
**CHRM4**	Cholinergic receptor, muscarinic 4	20%	4%	4.70
**COL23A1**	Collagen, type XXIII, alpha 1	20%	4%	4.70
**COL25A1**	Collagen, type XXV, alpha 1	20%	4%	5.65
**NDUFA9**	NADH-ubiquinone oxidoreductase 39 kDa subunit	20%	3%	7.06
**ORAI1**	Calcium release	20%	5%	4.03
**TIMM44**	Import inner membrane translocase subunit TIM44	20%	4%	5.65
**CCKBR**	Cholecystokinin B receptor	20%	4%	4.62
**CD33**	CD33 molecule	20%	1%	27.70
**CD36**	CD36 molecule (thrombospondin receptor)	20%	5%	3.96
**CD42B, GP1BA**	Glycoprotein Ib (platelet), alpha polypeptide	20%	1%	27.70
**CHRNB3**	Cholinergic receptor, nicotinic, beta	20%	3%	6.93
**CTNNBL1**	Beta catenin-like1	20%	6%	3.46
**ITGA8**	Integrin, alpha 8	20%	5%	3.96
**ANXA9**	AnnexinA9	20%	6%	3.40
**CHRNE**	Cholinergic receptor, nicotinic, epsilon	20%	5%	3.88
**DSG3-EC5**	Desmoglein 3	20%	5%	3.88
**EPS8L2**	EpidermalGrowth factor receptor pathway substrate 8-related protein 2	20%	6%	3.40
**CD81**	CD81 molecule	19%	4%	5.33
**ETFA**	Electron transfer flavoprotein alpha-subunit	19%	4%	4.44
**CYB5B**	Cytochrome b5 outer mitochondrial membrane isoform precursor	19%	1%	13.07
**FCGR2B**	Fc fragment of IgG, low affinity IIb, receptor	19%	5%	3.73
**PCDHB5**	Protocadherin beta 5 precursor	19%	4%	5.23
**SYNJ2BP**	Synaptojanin 2 binding protein	19%	3%	6.53
**ABAT**	4-aminobutyrate aminotransferase	19%	4%	4.27
**CHRNB2**	Cholinergic receptor, nicotinic, beta 2	19%	7%	2.85
**CHRNE**	Cholinergic receptor, nicotinic, epsilon	19%	5%	3.66
**DSG1**	Desmoglein 1	19%	7%	2.85
**GK2**	Glycerol kinase, testis specific 2	19%	5%	3.66
**MET-V1**	Homo sapiens mRNA for met proto-oncogene precursor variant protein.	19%	6%	3.20
**NDUFV3-V2**	NADH-ubiquinone oxidoreductase 9 kDa subunit	19%	4%	4.27
**AKAP10**	Protein kinase A Anchoring protein 10	18%	4%	5.02
**ATP2C1-V3**	ATPase, Ca++ transporting, type 2C, membe	18%	4%	5.02
**CHRNA4-V1**	Cholinergic receptor, nicotinic, alpha 4 subunit	18%	3%	6.27
**CD100, SEMA4D**	Sema domain, Ig domain, transmembrane domain and short cytoplasmic domain, (semaphorin) 4D	18%	4%	4.91
**CD34**	CD34 molecule	18%	6%	3.07
**CD69**	CD69 molecule	18%	5%	3.51
**CHRNA9**	Cholinergic receptor, nicotinic, alpha 9	18%	4%	4.09
**CHRNB4-V2**	Cholinergic receptor, nicotinic, beta 4	18%	5%	3.51
**CPT1B**	Carnitine O-palmitoyltransferase I, mitochondrial muscle isoform	18%	5%	3.51
**DSC1**	Desmocollin 1 isoform Dsc1a preproprotein	18%	7%	2.73
**EPS15L1**	EpidermalGrowth factor receptor pathway substrate 15-like 1	18%	6%	3.07
**HLA-C_V2**	Major histocompatibility complex, class I, C	18%	4%	4.91
**ITGB1**	Integrin beta 1 isoform 1C	18%	3%	6.14
**ME2**	NAD-dependent malic enzyme	18%	6%	3.07
**NDUFV3**	NADH-ubiquinone oxidoreductase 9 kDa subunit	18%	4%	4.91
**PGR**	Progesterone receptor	18%	6%	3.07

Suplemental Table 1. Reactivities of patient and control sera on protein micoarrays*

**CHRNB1**	Cholinergic receptor, nicotinic, beta 1 subunit	17%	5%	3.44
**CTNNBIP1**	Catenin, beta interacting protein 1	17%	4%	4.01
**ACTA1**	Actin, AlphA 1, skeletAl muscle	17%	6%	2.94
**CTNNAL1**	Catenin, alpha-like 1	17%	4%	4.70
**NDUFB10**	NADH-ubiquinone oxidoreductase PDSW subunit	17%	2%	7.84
**PCDHB15**	Protocadherin beta 15 precursor	17%	4%	3.92
**DSG1**	Desmoglein 1	17%	5%	3.36
**BDKRB2**	Bradykinin receptor B2	17%	4%	3.83
**CD19**	CD19 molecule	17%	5%	3.29
**CD3E**	CD3e molecule, epsilon (CD3-TCR complex)	17%	4%	3.83
**CD40_V2**	CD40 molecule, TNF receptor superfamily member 5	17%	6%	2.88
**CDH9**	Cadherin 9, type 2 preproprotein	17%	1%	11.50
**DSG1_EC3**	Desmoglein 1	17%	6%	2.88
**DSG3_EC1**	Desmoglein 3	17%	5%	3.29
**CD75, ST6GAL1**	ST6 beta-galactosamide alpha-2,6-sialyltranferase 1	16%	5%	3.21
**CDH17**	Cadherin 17 precursor	16%	3%	5.62
**NDUFB4**	NADH-ubiquinone oxidoreductase B15 subunit	16%	2%	7.49
**NR3C1**	Nuclear receptor subfamily 3, group C, member 1	16%	5%	3.21
**PMPCA**	Peptidase (mitochondrial processing) alpha	16%	4%	3.75
**SLC38A1**	Solute carrier family 38, member 1	16%	4%	3.75
**TIMM13**	Mitochondrial import inner membrane translocase subunit TIM13 B	16%	4%	4.50
**ATP2A2-V1**	ATPase, Ca++ transporting, cardiac muscle, slow twitch 2	16%	6%	2.74
**CACNA2D1**	CalciumChannel, voltagedependent, alpha	16%	3%	5.49
**CD3G**	CD3g molecule,Gamma (CD3-TCR complex)	16%	2%	7.32
**FGFR1**	FibroblastGrowth factor receptor 1	16%	4%	3.66
**GLUD2**	Glutamate dehydrogenase 2	16%	6%	2.74
**CD74**	CD74 molecule, major histocompatibility complex, class II invariant chain	16%	6%	2.68
**COL6A2**	Collagen, type VI, alpha 2	16%	2%	7.14
**GPAM**	Glycerol-3-phosphate acyltransferase	16%	6%	2.68
**HADH**	Short chain 3-hydroxyacyl-CoA dehydrogenase	16%	3%	5.36
**HMGCL**	Hydroxymethylglutaryl-CoA lyase	16%	5%	3.06
**PDGFRA**	Platelet-derivedGrowth factor receptor alpha	16%	5%	3.06
**AR**	Androgen receptor	15%	4%	4.18
**CD98, SLC7A5**	Solute carrier family 7 (cationic amino acid transporter, y+ system), member 5	15%	5%	2.99
**COL21A1**	Collagen, type XXI, alpha 1	15%	2%	6.97
**GRIA3**	Glutamate receptor, ionotrophic, AMPA3	15%	4%	3.48
**CD98, SLC3A2**	Solute carrier family 3 (activators of dibasic and neutral amino acid transport), member 2	15%	6%	2.55
**COL9A1**	Collagen, type IX, alpha 2	15%	4%	3.40
**COX10**	Protoheme IX farnesyltransferase	15%	2%	6.80
**CTNNA1**	Catenin, alpha 1	15%	5%	2.91
**HBE1**	Hemoglobin, epsilon 1	15%	4%	3.40
**PCDHB9**	Protocadherin beta 9 precursor	15%	5%	2.91
**TMLHE**	Trimethyllysine dioxygenase	15%	3%	5.10
**CD11C, ITGAX**	Integrin, alpha X (complement component 3 receptor 4 subunit)	14%	1%	9.93
**CD20, MS4A1**	Membrane-spanning 4-domains, subfamily A, member 1	14%	7%	2.21
**DSG1-EC2**	Desmoglein 1	14%	5%	2.84
**FECH**	Ferrochelatase	14%	3%	4.97
**HK2**	Hexokinase, type II	14%	7%	2.21
**MTX1**	Metaxin 1	14%	2%	6.62
**CD14**	CD14 molecule	14%	6%	2.42
**CD4**	CD4 molecule	14%	6%	2.42
**CD66e,CEACAM5**	Carcinoembryonic antigen-related cell adhesion molecule 5	14%	5%	2.76
**DSC2**	Desmocollin 2 isoform Dsc2a preproprotein	14%	6%	2.42
**FCRLB**	Fc receptor-like B	14%	4%	3.22

Suplemental Table 1. Reactivities of patient and control sera on protein micoarrays*

**MUPCDH**	Mucin and cadherin-like isoform 1	14%	6%	2.42
**NDUFA2**	NADH-ubiquinone oxidoreductase B8 subunit	14%	3%	4.84
**SDHA**	Succinate dehydrogenase (ubiquinone) flavoprotein subunit	14%	6%	2.42
**SLC7A8**	solute carrier family 7 (cationic amino acid	14%	4%	3.87
**CKMT1B**	Creatine kinase	14%	3%	4.70
**COX17**	Cytochrome c oxidase copper chaperone	14%	3%	4.70
**NDUFA12**	NADH dehydrogenase [ubiquinone] 1 alpha subcomplex subunit 12;	14%	7%	2.09
**OGFRL1**	OpioidGrowth factor receptor-like 1	14%	4%	3.76
**ANXA9**	AnnexinA9	13%	4%	3.05
**CHRM1**	Cholinergic receptor, muscarinic 1	13%	3%	4.57
**COL9A1**	Collagen, type IX, alpha 1	13%	6%	2.29
**CPT1A**	Carnitine O-palmitoyltransferase I, mitochondrial liver isoform	13%	4%	3.05
**HLA-A**	Major histocompatibility complex, class I, A	13%	6%	2.29
**HLA-DPB1**	Major histocompatibility complex, class II, DP beta 1	13%	7%	2.03
**ATP2A2-V1**	ATPase, Ca++ transporting, cardiac muscle, slow twitch 2	13%	6%	2.22
**BDKRB1**	Bradykinin receptor B1	13%	7%	1.97
**CD3D**	CD3d molecule, delta (CD3-TCR complex)	13%	5%	2.54
**CD42a, GP9, GPIX**	Glycoprotein IX (platelet)	13%	4%	3.55
**DSG3_S2**	Desmoglein 3	13%	4%	2.96
**TIMM9**	Mitochondrial import inner membrane translocase subunit TIM9 A	13%	7%	1.97
**CACNB4**	CalciumChannel, voltagedependent, beta 4	13%	4%	2.88
**CHRNA2**	Cholinergic receptor, nicotinic, alpha 2	13%	5%	2.46
**CHRNA7**	Cholinergic receptor, nicotinic, alpha 7	13%	3%	4.31
**COX5B**	Cytochrome c oxidase polypeptide Vb	13%	4%	2.88
**DSG2**	Desmoglein 2	13%	5%	2.46
**PDK1**	Pyruvate dehydrogenase [lipoamide]] kinase isozyme 1	13%	4%	3.45
**PIGR**	Polymeric immunoglobulin receptor	13%	3%	4.31
**SDHD**	Succinate dehydrogenase (ubiquinone) cytochrome B small subunit	13%	2%	5.75
**SLC36A4**	Solute carrier family 36, member 4	13%	1%	17.25
**ATP2A2-V2**	ATPase, Ca++ transporting, cardiac muscle, slow twitch 2	12%	4%	2.79
**CD99**	CD99 molecule	12%	4%	3.35
**NDUFA4**	NADH-ubiquinone oxidoreductase MLRQ subunit	12%	3%	4.18
**ABCB7**	ATP-binding cassette transporter 7	12%	5%	2.31
**CD10, MME**	Membrane metallo-endopeptidase	12%	3%	4.05
**CD11A, ITGAL-2**	Integrin, alpha L (antigen CD11A (p180), lymphocyte function-associated antigen 1; alpha polypeptide)	12%	1%	8.10
**CD71, TFRC**	Transferrin receptor (p90, CD71)	12%	3%	4.05
**CD8B**	CD8b molecule	12%	7%	1.80
**CD96**	CD96 molecule	12%	6%	2.03
**COL20A1**	Collagen, type XX, alpha 1	12%	4%	3.24
**FCRL5**	Fc receptor-like 5	12%	5%	2.31
**HLA-DRB1**	Major histocompatibility complex, class II, DR beta 1	12%	5%	2.31
**ITGA5**	IntegrIn alpha 5 precursor	12%	4%	2.70
**MAOA**	Amine oxidase (flavin-containing) A	12%	4%	2.70
**NDUFS1**	NADH-ubiquinone oxidoreductase 75 kDa subunit	12%	1%	16.20
**PCDH8**	Protocadherin 8 isoform 1 precursor	12%	4%	3.24
**PCDHB10**	Protocadherin beta 10 precursor	12%	6%	2.03
**SUOX**	Sulfite oxidase	12%	5%	2.31
**CD13, ANPEP**	Alanyl (membrane) aminopeptidase	11%	4%	3.14
**MCCC2**	Methylcrotonyl-CoA carboxylase beta chain	11%	4%	3.14
**ATP2A2-V2**	ATPase, Ca++ transporting, cardiac muscle, slow twitch 2	11%	5%	2.17
**CD207**	CD207 molecule, langerin	11%	7%	1.68
**COL3A1**	Collagen, type III, alpha 1	11%	4%	2.53
**NDUFB2**	NADH-ubiquinone oxidoreductase AGGG subunit	11%	5%	2.17
**SLC16A10**	Solute carrier family 16, member 10	11%	1%	7.58

Suplemental Table 1. Reactivities of patient and control sera on protein micoarrays*

**CD1D**	CD1d molecule	11%	4%	2.93
**CD2**	CD2 molecule	11%	6%	1.83
**COX11**	Cytochrome c oxidase assembly homolog	11%	4%	2.93
**DHODH**	Dihydroorotate dehydrogenase	11%	4%	2.93
**GK-V1**	Glycerol kinase, testis specific 1	11%	4%	2.93
**CACNG7**	Voltagedependent calcium channelGamma7	10%	3%	3.53
**CD36**	CD36 molecule (thrombospondin receptor)	10%	4%	2.35
**CDH13**	Cadherin 13 preproprotein	10%	3%	3.53
**CDS2**	Phosphatidate cytidylyltransferase 2	10%	4%	2.82
**DSG3_EC2**	Desmoglein 3	10%	7%	1.57
**EPS8L1-V1**	EPS8-like 1, isoform a	10%	4%	2.35
**GOT1**	Glutamic-oxaloacetic transaminase 1	10%	6%	1.76
**HLA-B**	Major histocompatibility complex, class I, B	10%	4%	2.35
**ITGB2**	Integrin, beta 2 precursor	10%	7%	1.57
**UCP2**	Mitochondrial uncoupling protein 2 (UCP 2) (UCPH)	10%	5%	2.02
**APLNR**	Apelin receptor	10%	4%	2.72
**CD50, ICAM3**	Intercellular adhesion molecule 3	10%	4%	2.72
**GOT2**	Glutamic-oxaloacetic transaminase 2	10%	6%	1.70
**SLC25A5**	solute carrier family 25 (mitochondrial carrier; citrate transporter), member 5	10%	4%	2.27
**CD18, ITGB2**	integrin, beta 2 (complement component 3 receptor 3 and 4 subunit)	9%	6%	1.63
**CD1B**	CD1b molecule	9%	1%	13.07
**COL1A2**	Collagen, type I, alpha 2	9%	5%	1.87
**DSG3_S1**	Desmoglein 3	9%	4%	2.18
**DSG3**	Desmoglein 3	9%	6%	1.63
**DSG4**	Desmoglein 4 isoform 2	9%	5%	1.87
**F2RL1**	Coagulation factor II (thrombin) receptorlike 1	9%	4%	2.18
**GRIN2A**	Glutamate receptor, ionotropic, N-methyl D-aspartate 2A	9%	4%	2.18
**ITGB6**	Integrin, beta 6	9%	3%	3.27
**MTRR-V1**	Methionine synthase reductase	9%	4%	2.18
**NDUFV1**	NADH-ubiquinone oxidoreductase 51 kDa subunit	9%	1%	6.53
**UPK3B**	Uroplakin 3B isoform a	9%	7%	1.45
**CD92, SLC44A1**	Solute carrier family 44, member 1	9%	2%	4.18
**CRHR1**	Corticotropin releasing hormone receptor 1	9%	3%	3.14
**FACL6**	Acyl-CoA synthetase long-chain family member 6	9%	2%	4.18
**CACNA1G-S2**	VoltagedependentCalciumChannel alpha 1G	9%	4%	2.40
**CD69**	CD69 molecule	9%	1%	6.01
**CTNNA2**	Catenin, alpha 2	9%	4%	2.00
**FGFRL1**	FibroblastGrowth factor receptor-like 1	9%	4%	2.00
**MTRR-V2**	Methionine synthase reductase	9%	5%	1.72
**PKP4**	Plakophilin 4 isoform a	9%	4%	2.00
**C1QBP**	Complement component 1, q subcomponent binding protein	8%	4%	2.30
**CD5**	CD5 molecule	8%	2%	3.83
**CD57, B3GAT1**	Beta-1,3-glucuronyltransferase 1 (glucuronosyltransferase P)	8%	4%	2.30
**DSG1**	Desmoglein 1	8%	2%	3.83
**ORAI1**	Calcium release	8%	3%	2.88
**PMPCB**	Mitochondrial processing peptidase beta subunit	8%	4%	1.92
**SLC25A17**	Solute carrier family 25 (mitochondrial carrier; peroxisomal membrane protein, 34kDa), member 17	8%	1%	5.75
**CD41, ITGA2B-2**	Integrin, alpha 2b (plateletGlycoprotein IIb of IIb/IIIa complex, antigen CD41)	8%	4%	1.83
**CD66b, CEACAM8**	Carcinoembryonic antigen-related cell adhesion molecule 8	8%	5%	1.57
**CHRND**	Cholinergic receptor, nicotinic, delta	8%	3%	2.74
**COQ3**	Hexaprenyldihydroxybenzoate methyltransferase	8%	3%	2.74
**ECHS1**	Enoyl-CoA hydratase	8%	4%	2.20
**HRH2**	Histamine receptor H2	8%	4%	2.20
**HTRA2**	HtrA serine peptidase 2	8%	4%	1.83

Suplemental Table 1. Reactivities of patient and control sera on protein micoarrays*

**NDUFS6**	NADH-ubiquinone oxidoreductase 13 kDa-A subunit	8%	4%	2.20
**UCRC**	Ubiquinol-cytochrome c reductase, complex III subunit X	8%	5%	1.57
**CACNA1G-S3**	VoltagedependentCalciumChannel alpha 1G	8%	6%	1.31
**CDH11**	Cadherin 11, type 2 preproprotein	8%	4%	1.74
**NDUFA7**	NADH-ubiquinone oxidoreductase subunit B14.5a	8%	4%	2.09
**NGFRAP1**	NerveGrowth factor receptor (TNFRSF16)	8%	4%	2.09
**UQCRQ**	Ubiquinol-cytochrome c reductase, complex III subunit VII	8%	5%	1.49
**CD52**	CD52 molecule	7%	1%	4.97
**CYCS**	Cytochrome c	7%	4%	1.66
**DSC3**	Desmocollin 3 isoform Dsc3a preproprotein	7%	9%	0.83
**EPS8L3**	EpidermalGrowth factor receptor pathway substrate 8-related protein 3	7%	4%	1.99
**MIPEP**	Mitochondrial intermediate peptidase	7%	1%	9.93
**NDUFV2**	NADH-ubiquinone oxidoreductase 24 kDa subunit	7%	4%	1.99
**SLC9A6**	Sodium/hydrogen exchanger 6 (Na(+)/H(+) exchanger 6) (NHE-6)	7%	4%	1.99
**BAD**	Bcl2-antagonist of cell death (BAD)	7%	4%	1.88
**CACNA1S-S3**	CalciumChannel, voltagedependent, L type,	7%	4%	1.88
**DSG2**	Desmoglein 2	7%	7%	1.05
**DSG3_EC5**	Desmoglein 3	7%	4%	1.57
**IGF1R**	Insulin-likeGrowth factor 1 receptor precursor	7%	1%	4.70
**PPIF**	Peptidyl-prolyl cis-trans isomerase	7%	8%	0.86
**ACTG2**	Actin,GAmmA 2, smooth muscle, enteric	6%	4%	1.48
**CHRNA2**	Cholinergic receptor, nicotinic, alpha 2	6%	4%	1.78
**DSG1**	Desmoglein 1	6%	5%	1.27
**FLT3LG**	Fms-related tyrosine kinase 3 ligand	6%	4%	1.48
**MTX2-V1**	Metaxin 2	6%	4%	1.48
**NDUFV3-V1**	NADH-ubiquinone oxidoreductase 9 kDa subunit	6%	5%	1.27
**AGTR1**	Angiotensin II receptor, type 1	6%	4%	1.39
**CD29, ITGB2**	Integrin, beta 1 (fibronectin receptor, beta polypeptide, antigen CD29 includes MDF2, MSK12)	6%	2%	2.79
**CD3G**	CD3g molecule,Gamma (CD3-TCR complex)	6%	4%	1.39
**CHRNA10**	Cholinergic receptor, nicotinic, alpha 10	6%	3%	2.09
**FCGRT**	FcFragment of IgG, receptor, transporter,	6%	5%	1.19
**FPGS**	Folylpolyglutamate synthase	6%	2%	2.79
**NDUFA1**	NADH-ubiquinone oxidoreductase MWFE subunit	6%	3%	2.09
**PTH1R**	Parathyroid hormone 1 receptor	6%	3%	2.09
**SCP3**	Synaptonemal complex protein 3	6%	4%	1.39
**CD47**	CD47 molecule	6%	5%	1.12
**CD8A**	CD8a molecule	6%	6%	0.98
**CD97**	CD97 molecule	6%	7%	0.87
**CKMT2**	Creatine kinase	6%	2%	2.61
**TIMM22**	Mitochondrial import inner membrane translocase subunit TIM22	6%	1%	3.92
**UQCRFS1**	Ubiquinol-cytochrome C reductase iron-sulfur subunit	6%	4%	1.31
**CD27**	CD27 molecule	5%	5%	1.05
**CD58**	CD58 molecule	5%	4%	1.46
**CD9**	CD9 molecule	5%	4%	1.46
**DSC3**	Desmocollin 3 isoform Dsc3a preproprotein	5%	6%	0.91
**GCDH-V1**	Glutaryl-CoA dehydrogenase	5%	4%	1.22
**HSPD1**	Heat shock 60kDa protein 1	5%	4%	1.46
**ITGB1BP2**	Integrin beta 1 binding protein 2	5%	4%	1.46
**TSPO**	translocator protein	5%	2%	2.44
**UQCRC2**	Ubiquinol-cytochrome C reductase complex core protein 2	5%	5%	1.05
**ANXA4**	Annexin IV	5%	6%	0.85
**CD1A**	CD1a molecule	5%	7%	0.76
**CD28**	CD28 molecule	5%	6%	0.85
**CD55**	CD55 molecule, decay accelerating factor for complement (Cromer bloodGroup)	5%	4%	1.13

Suplemental Table 1. Reactivities of patient and control sera on protein micoarrays*

**CD66d,CEACAM3**	Carcinoembryonic antigen-related cell adhesion molecule 3	5%	4%	1.13
**CDH16**	Cadherin 16 precursor	5%	2%	2.27
**CDH24**	Cadherin	5%	4%	1.36
**CHRM2**	Cholinergic receptor, muscarinic 2	5%	6%	0.85
**COL5A1**	Collagen, type V, alpha 1	5%	4%	1.36
**COLQ**	Collagen-like tail subunit	5%	4%	1.13
**FCRL4**	Fc receptor-like 4	5%	4%	1.36
**NDUFA13**	NADH dehydrogenase [ubiquinone] 1 alpha subcomplex subunit 13	5%	5%	0.97
**NDUFV1-V1**	NADH-ubiquinone oxidoreductase 51 kDa subunit	5%	1%	3.40
**SLC25A15**	Solute carrier family 25 (aspartate/glutamate carrier), member 15	5%	3%	1.70
**SLC25A19**	Solute carrier family 25, member 19	5%	4%	1.13
**SLC25A39**	Solute carrier family 25, member 39	5%	6%	0.85
**SLC36A2**	Solute carrier family 36, member 2	5%	2%	2.27
**TACR1**	Tachykinin receptor 1	5%	4%	1.13
**WARS2**	Tryptophanyl-tRNA synthetase	5%	1%	6.80
**CD19**	CD19 molecule	5%	1%	6.27
**CD66f, PSG1**	Pregnancy specific beta-1-glycoprotein 1	5%	2%	2.09
**HLA-DQA1**	Major histocompatibility complex, class II, DQ alpha 1	5%	7%	0.63
**ITGB1BP1**	Integrin cytoplasmic domain-associated protein 1	5%	3%	1.57
**OXA1L**	Cytochrome oxidase biogenesis protein OXA1	5%	5%	0.90
**PCCA**	Propionyl-CoA carboxylase alpha chain	5%	7%	0.70
**PCDH20**	Protocadherin 20	5%	4%	1.25
**SLC25A20**	Solute carrier family 25 (mitochondrial carrier; ornithine transporter) member 20	5%	1%	6.27
**AKAP1**	Protein kinase A Anchoring protein 1	4%	4%	1.15
**ANXA8L2**	AnnexinA8L2	4%	4%	0.96
**BCL2**	Apoptosis regulator Bcl-2	4%	1%	2.88
**CD40**	CD40 molecule, TNF receptor superfamily member 5	4%	5%	0.82
**CD79**	CD79b molecule, immunoglobulin-associated beta	4%	5%	0.82
**CD83**	CD83 molecule	4%	6%	0.72
**CD89, FCAR**	Fc fragment of IgA, receptor for	4%	4%	0.96
**CD90, THY1**	Thy-1 cell surface antigen	4%	4%	0.96
**CD94, KLRD1**	Killer cell lectin-like receptor subfamily D, member 1	4%	4%	1.15
**CHRNA10**	Cholinergic receptor, nicotinic, alpha 10	4%	4%	1.15
**COX6A1**	Cytochrome c oxidase polypeptide VIa-liver	4%	7%	0.58
**CS**	Citrate synthase	4%	1%	2.88
**FLT4**	Fms-related tyrosine kinase 4	4%	6%	0.72
**ITGAV**	Integrin alpha-V isoform 1 precursor	4%	6%	0.72
**NDUFB1**	NADH-ubiquinone oxidoreductase MNLL subunit	4%	5%	0.82
**PCCB**	Propionyl-CoA carboxylase beta chain	4%	4%	0.96
**SCP2**	Sterol carrier protein 2	4%	6%	0.72
**TOMM40**	Probable mitochondrial import receptor subunit TOM40 homolog	4%	2%	1.92
**ACTC1**	Actin, AlphA, cArdiAc muscle 1	4%	4%	1.05
**CD24**	CD24 molecule	4%	4%	0.87
**CD73, NT5E**	5'-nucleotidase, ecto (CD73)	4%	4%	0.87
**CD86**	CD86 molecule	4%	5%	0.75
**CDH7**	Cadherin 7, type 2 preproprotein	4%	4%	1.05
**CHRNA10**	Cholinergic receptor, nicotinic, alpha 10	4%	2%	1.74
**CHRNA9**	Cholinergic receptor, nicotinic, alpha 9	4%	7%	0.58
**HLA-F**	Major histocompatibility complex, class I, F	4%	7%	0.52
**IGF2R**	Insulin-likeGrowth factor 1 receptor precursor	4%	4%	0.87
**IGFBP2**	Insulin-likeGrowth factor 2 mRNA binding protein 2	4%	3%	1.31
**MDH2**	Malate dehydrogenase	4%	5%	0.75
**NDUFB5**	NADH-ubiquinone oxidoreductase SGDH subunit	4%	3%	1.31
**NDUFS4**	NADH-ubiquinone oxidoreductase 18 kDa subunit	4%	6%	0.65

Suplemental Table 1. Reactivities of patient and control sera on protein micoarrays*

**SLC25A14**	Solute carrier family 25 (aspartate/glutamate carrier), member 14	4%	4%	0.87
**UPK2**	Uroplakin 2	4%	3%	1.31
**UQCRB-V1**	Ubiquinol-cytochrome C reductase complex 14 kDa protein, isoform 1	4%	4%	0.87
**VDAC2**	Voltage-dependent anion-selective channel protein 2	4%	2%	1.74
**AK2**	Adenylate kinase isoenzyme 2	3%	3%	1.18
**ANXA1**	Annexin I	3%	7%	0.52
**ATP5O**	ATP synthase oligomycin sensitivity conferral protein	3%	6%	0.59
**BCKDK-V2**	Branched chain ketoacid dehydrogenase kinase , isoform b	3%	1%	4.70
**CD5**	CD5 molecule	3%	5%	0.67
**CDH20**	Cadherin 20, type 2 preproprotein	3%	5%	0.67
**CHRNA6**	Cholinergic receptor, nicotinic, alpha 6	3%	7%	0.52
**DSC1**	Desmocollin 1 isoform Dsc1a preproprotein	3%	5%	0.67
**DSG1_EC4**	Desmoglein 1	3%	5%	0.67
**FCRL2**	Fc receptor-like 2	3%	1%	4.70
**GPX4**	Phospholipid hydroperoxideGlutathione peroxidase	3%	6%	0.59
**HLA-C-V1**	Major histocompatibility complex, class I, C	3%	6%	0.59
**HSPE1**	Heat shock 10kDa protein 1	3%	7%	0.52
**IGF2**	Insulin-likeGrowth factor 2	3%	2%	1.57
**LARS2**	Probable leucyl-tRNA synthetase	3%	4%	0.78
**MTX2-V2**	Metaxin 2	3%	3%	1.18
**PCDHB1**	Protocadherin beta 1 precursor	3%	7%	0.52
**TOMM70A**	Mitochondrial precursor proteins import receptor (Translocase of outermembrane TOM70)	3%	6%	0.59
**CACNA1G-S2**	VoltagedependentCalciumChannel alpha 1G	3%	4%	0.84
**CD68**	CD68 molecule	3%	7%	0.46
**CHRNB3**	Cholinergic receptor, nicotinic, beta	3%	4%	0.84
**COX4I1**	Cytochrome c oxidase subunit IV isoform 1	3%	7%	0.46
**CTNNA3-V1**	Catenin, alpha 3	3%	4%	0.84
**DLAT**	Dihydrolipoamide acetyltransferase component of pyruvate dehydrogenasecomplex	3%	1%	4.18
**DSG1**	Desmoglein 1	3%	1%	2.09
**HLA-DQB1**	Major histocompatibility complex, class II, DQ beta 1	3%	4%	0.84
**HLCS**	Biotin-protein ligase	3%	6%	0.52
**MCCC1**	Methylcrotonyl-CoA carboxylase alpha chain	3%	5%	0.60
**SLC25A12**	Solute carrier family 25 (aspartate/glutamate carrier), member 12	3%	5%	0.60
**TOMM20**	Mitochondrial import receptor subunit TOM20 homolog	3%	7%	0.46
**ANXA6**	Annexin VI isoform 1	3%	4%	0.61
**CACNA1S-S2**	CalciumChannel, voltagedependent, L type,	3%	1%	3.66
**CD43, SPN**	Sialophorin	3%	4%	0.61
**CD80**	CD80 molecule	3%	4%	0.61
**CD85, LILRB1**	Leukocyte immunoglobulin-like receptor, subfamily B (with TM and ITIM domains), member 1	3%	2%	1.22
**CHRNB4-V2**	Cholinergic receptor, nicotinic, beta 4	3%	2%	1.22
**COL4A3BP**	Collagen, type IV, alpha 3 (Goodpasture antigen) binding protein	3%	4%	0.73
**COL9A3**	Collagen, type IX, alpha 3	3%	3%	0.91
**COX6A2**	Cytochrome c oxidase polypeptide VIa-heart	3%	8%	0.33
**DSG3_A53625-88**	Desmoglein 3	3%	8%	0.33
**DSG4**	Desmoglein 4 isoform 2	3%	5%	0.52
**GRB7**	Growth factor receptor-bound protein 7	3%	7%	0.41
**ITGAX**	Integrin alpha X precursor	3%	5%	0.52
**MYCBP**	C-Myc binding protein	3%	5%	0.52
**NDUFS3**	NADH-ubiquinone oxidoreductase 30 kDa subunit	3%	3%	0.91
**PCDHB11**	Protocadherin beta 11 precursor	3%	2%	1.22
**PCDHB13**	Protocadherin beta 13 precursor	3%	4%	0.73
**PDHX**	pyruvate dehydrogenase complex,ccomponent X	3%	7%	0.37
**PERP**	PerP, TP53 apoptosis effector	3%	4%	0.61
**PERP**	PerP, TP53 apoptosis effector	3%	5%	0.52

Suplemental Table 1. Reactivities of patient and control sera on protein micoarrays*

**SLC25A17**	Solute carrier family 25 (mitochondrial carrier; peroxisomal membrane protein, 34kDa), member 17	3%	3%	0.91
**TIMM17B**	Mitochondrial import inner membrane translocase subunit TIM17 B (JM3)	3%	4%	0.61
**TIMM8B**	Mitochondrial import inner membrane translocase subunit TIM8 B	3%	5%	0.52
**CACNG3**	Voltagedependent calcium channelGamma3	2%	4%	0.52
**CD1C**	CD1c molecule	2%	3%	0.78
**CD7**	CD7 molecule	2%	6%	0.39
**CD70**	CD70 molecule	2%	6%	0.39
**CD95, FAS**	Fas (TNF receptor superfamily, member 6)	2%	4%	0.52
**CDH2**	Cadherin 2, type 1 preproprotein	2%	4%	0.52
**CHRFAM7A**	CHRNA7FAM7A fusion isoform 1	2%	4%	0.52
**CHRM1**	Cholinergic receptor, muscarinic 1	2%	3%	0.78
**CHRM5**	Cholinergic receptor, muscarinic 5	2%	5%	0.45
**CHRNA3**	Cholinergic receptor, nicotinic, alpha 3	2%	4%	0.63
**CKMT2**	Creatine kinase	2%	7%	0.35
**CPT2**	Carnitine O-palmitoyltransferase II, mitochondrial precursor	2%	7%	0.31
**CTNNB1**	Catenin (cadherin-associated protein), beta 1,	2%	5%	0.45
**DIABLO**	IAP-binding mitochondrial protein	2%	1%	1.57
**DSG2**	Desmoglein 2	2%	1%	3.14
**DSG3**	Desmoglein 3	2%	7%	0.35
**GATM**	Glycine amidinotransferase	2%	1%	3.14
**GPT**	Alanine aminotransferase	2%	4%	0.52
**HRH1**	Histamine receptor H1	2%	4%	0.63
**ITGAL**	Integrin alpha L isoform a precursor	2%	3%	0.78
**NNT-V2**	NAD(P) transhydrogenase	2%	4%	0.63
**OXCT1**	Succinyl-CoA:3-ketoacid-coenzyme A transferase	2%	4%	0.63
**PCDH21**	Protocadherin 21 precursor	2%	6%	0.39
**PDGFRL**	Platelet-derivedGrowth factor receptor	2%	4%	0.63
**SLC25A1**	solute carrier family 25 (mitochondrial carrier; citrate transporter), member 1	2%	4%	0.63
**SLC25A4**	solute carrier family 25 (mitochondrial carrier; citrate transporter), member 4	2%	4%	0.63
**ACTB**	Actin, betA	2%	4%	0.52
**ACTG1**	Actin,GAmmA 1	2%	3%	0.65
**AGTRAP**	Angiotensin II receptor, type 2	2%	3%	0.65
**AK3**	GTP:AMP phosphotransferase	2%	7%	0.29
**ALAS1**	Aminolevulinate, delta-, synthase 1	2%	4%	0.44
**ATP2C1-V2**	ATPase, Ca++ transporting, type 2C, membe	2%	6%	0.33
**BCAT2**	Branched-chain amino acid aminotransferase	2%	6%	0.33
**BCKDHB**	Branched chain keto acid dehydrogenase E1, beta polypeptide	2%	4%	0.44
**C5orf39**	Annexin II receptor	2%	3%	0.65
**CACNB1**	CalciumChannel, voltagedependent, beta 1	2%	1%	2.61
**CACNG1**	Voltagedependent calcium channelGamma1	2%	2%	0.87
**CACNG4**	Voltagedependent calcium channelGamma4	2%	4%	0.44
**CD83**	CD83 molecule	2%	5%	0.37
**CD9**	CD9 molecule	2%	4%	0.44
**CDH19**	Cadherin 19, type 2 preproprotein	2%	4%	0.52
**CDH23**	Cadherin	2%	4%	0.52
**CHRNA1-V1**	Cholinergic receptor, nicotinic, alpha 1isoform b	2%	4%	0.44
**CHRNA4-V2**	Cholinergic receptor, nicotinic, alpha 4 subunit	2%	5%	0.37
**CHRNA6**	Cholinergic receptor, nicotinic, alpha 6	2%	6%	0.33
**COL10A1**	Collagen, type X, alpha 1	2%	4%	0.44
**COL19A1**	Collagen, type XIX, alpha 1	2%	4%	0.44
**COL4A6**	Collagen, type IV, alpha 6	2%	6%	0.33
**COX6C**	Cytochrome c oxidase polypeptide VIc precursor	2%	5%	0.37
**CTNND1-V1**	Catenin, delta 1 isoform 1ABC	2%	3%	0.65
**DBT**	Lipoamide acyltransferase component of branched-chain alpha-keto aciddehydrogenase complex subunit)	2%	5%	0.37

Suplemental Table 1. Reactivities of patient and control sera on protein micoarrays*

**DNAJA3**	DnaJ (Hsp40) homolog, subfamily A, member 3	2%	7%	0.26
**DSC2**	Desmocollin 2 isoform Dsc2a preproprotein	2%	6%	0.33
**DSG3_+A697S2**	Desmoglein 3	2%	7%	0.26
**DSG4**	Desmoglein 4 isoform 2	2%	3%	0.65
**F2RL2**	Coagulation factor II (thrombin) receptorlike 2	2%	4%	0.44
**FDX1**	Adrenodoxin, mitochondrial precursor	2%	5%	0.37
**GRB2**	Growth factor receptor-bound protein 2 isoform	2%	4%	0.52
**HCCS**	Cytochrome c-type heme lyase	2%	5%	0.37
**HLA-DRA**	Major histocompatibility complex, class II, DR alpha	2%	4%	0.52
**HRH4**	Histamine H4 receptor isoform 1	2%	5%	0.37
**ITGB8**	Integrin, beta 8	2%	4%	0.44
**NDUFA5**	NADH-ubiquinone oxidoreductase 13 kDa-B subunit	2%	6%	0.33
**NDUFS2**	NADH-ubiquinone oxidoreductase 49 kDa subunit	2%	4%	0.44
**NFS1**	Nitrogen fixation 1 homolog	2%	1%	2.61
**NFS2**	Nitrogen fixation 2 homolog	2%	1%	2.61
**PCDHB14**	Protocadherin beta 14 precursor	2%	4%	0.44
**PERP**	PerP, TP53 apoptosis effector	2%	5%	0.37
**PERP**	PerP, TP53 apoptosis effector	2%	4%	0.44
**PPOX**	Protoporphyrinogen oxidase	2%	3%	0.65
**SHMT1**	Serine hydroxymethyltransferase, cytosolic	2%	4%	0.52
**SLC25A10**	solute carrier family 25 (mitochondrial carrier; citrate transporter), member 10	2%	3%	0.65
**SLC25A11**	solute carrier family 25 (mitochondrial carrier; citrate transporter), member 11	2%	1%	1.31
**SLC25A31**	Solute carrier family 25 (mitochondrial carrier; adenine nucleotide translocator), member 31	2%	4%	0.52
**SLC6A6-V3**	solute carrier family 6 (neurotransmiter)	2%	5%	0.37
**SURF1**	Surfeit locus protein 1	2%	5%	0.37
**TIMM23**	Mitochondrial import inner membrane translocase subunit TIM23	2%	7%	0.29
**TK2**	Thymidine kinase 2, mitochondrial	2%	6%	0.33
**TST**	Thiosulfate sulfurtransferase (Rhodanese)	2%	6%	0.33
**UCRC**	Ubiquinol-cytochrome c reductase, complex III subunit X	2%	5%	0.37
**ABCB6**	ATP-binding cassette transporter 6	2%	6%	0.26
**ABCD4**	ATP-binding cassette, sub-family D (ALD), member 4	2%	6%	0.26
**ACADS**	Acyl-CoA dehydrogenase, short-chain specific	2%	4%	0.42
**ACADSB**	Acyl-CoA dehydrogenase, short/branched chain specific	2%	4%	0.35
**ACSL3**	Acyl-CoA synthetAse long-chAin fAmily member 3	2%	4%	0.35
**ACTN2**	Actinin, AlphA 2	2%	5%	0.30
**AK3L1**	Adenylate kinase 3	2%	2%	0.70
**ATP2C1-V1**	ATPase, Ca++ transporting, type 2C, membe	2%	4%	0.35
**ATP5A1**	ATP synthase alpha chain	2%	4%	0.42
**CACNA1G-S1**	VoltagedependentCalciumChannel alpha 1G	2%	6%	0.26
**CD72**	CD72 molecule	2%	5%	0.30
**CDH12**	Cadherin 12, type 2 preproprotein	2%	4%	0.42
**CHRFAM7A**	CHRNA7FAM7A fusion isoform 1	2%	4%	0.35
**CKMT1A**	Creatine kinase	2%	4%	0.35
**COL20A1**	Collagen, type XX, alpha 1	2%	5%	0.30
**COL6A1**	Collagen, type VI, alpha 1	2%	5%	0.30
**DLD**	Dihydrolipoamide dehydrogenase	2%	3%	0.52
**DSG1_EC1**	Desmoglein 1	2%	7%	0.23
**DSG3_EC4**	Desmoglein 3	2%	5%	0.30
**DSG3-EC1**	Desmoglein 3	2%	5%	0.30
**GRIA2**	Glutamate receptor, ionotropic, AMPA2	2%	3%	0.52
**HADHA**	Trifunctional enzyme alpha subunit	2%	5%	0.30
**HK1-V4**	Hexokinase, type I	2%	3%	0.52
**ITGB1BP3**	Integrin beta 1 binding protein 3	2%	4%	0.42
**JUP-V1**	Junction plakoglobin	2%	5%	0.30

Suplemental Table 1. Reactivities of patient and control sera on protein micoarrays*

**MLYCD**	Malonyl-CoA decarboxylase	2%	4%	0.35
**NDUFA10**	NADH-ubiquinone oxidoreductase 42 kDa subunit	2%	3%	0.52
**PCDHB16**	Protocadherin beta 16 precursor	2%	4%	0.35
**PERP**	PerP, TP53 apoptosis effector	2%	4%	0.42
**PKP2**	Plakophilin 2 isoform 2a	2%	7%	0.21
**SLC25A18**	Solute carrier family 25, member 18	2%	4%	0.42
**SLC25A2**	Solute carrier family 25 (mitochondrial carrier; ornithine transporter) member 2	2%	3%	0.52
**SLC7A13**	solute carrier family 7, (cationic amino acid	2%	6%	0.26
**TOMM40**	Probable mitochondrial import receptor subunit TOM40 homolog	2%	4%	0.35
**UPK1B**	Uroplakin 1B	2%	4%	0.35
**VDAC3**	Voltage-dependent anion-selective channel protein 3	2%	3%	0.52
**YWHAE**	Tyrosine 3-monooxygenase/tryptophan 5-monooxygenase activation protein, epsilon polypeptide	2%	4%	0.35
**ACADM-V3**	Acyl-CoA dehydrogenase, medium-chain specific	1%	1%	1.57
**ANXA13**	AnnexinA13 isoform b	1%	1%	0.78
**ATP2C1-V3**	ATPase, Ca++ transporting, type 2C, membe	1%	6%	0.20
**ATP7B**	Copper-transporting ATPase 2	1%	4%	0.31
**CCKAR**	Cholecystokinin A receptor	1%	1%	0.78
**CD1A**	CD1a molecule	1%	4%	0.26
**CD38**	CD38 molecule	1%	5%	0.22
**CD66a, CEACAM1**	Carcinoembryonic antigen-related cell adhesion molecule 1 (biliaryGlycoprotein)	1%	6%	0.20
**CD70**	CD70 molecule	1%	5%	0.22
**CD82**	CD82 molecule	1%	5%	0.22
**CD84**	CD84 molecule	1%	6%	0.20
**CD8B**	CD8b molecule	1%	5%	0.22
**CHRNA3**	Cholinergic receptor, nicotinic, alpha 3	1%	3%	0.39
**CHRNA4-V1**	Cholinergic receptor, nicotinic, alpha 4 subunit	1%	1%	0.78
**CHRNA4-V2**	Cholinergic receptor, nicotinic, alpha 4 subunit	1%	1%	0.78
**CHRNA7**	Cholinergic receptor, nicotinic, alpha 7	1%	3%	0.39
**CHRNG**	Cholinergic receptor, nicotinic,Gamma	1%	1%	0.78
**CKMT1A**	Creatine kinase	1%	1%	0.78
**CTNND2**	Catenin (cadherin-associated protein), delta 1,	1%	7%	0.16
**DSC3**	Desmocollin 3 isoform Dsc3a preproprotein	1%	7%	0.17
**DSG1_1-496**	Desmoglein 1	1%	6%	0.20
**DSG3**	Desmoglein 3	1%	1%	1.57
**DSG3_EC3**	Desmoglein 3	1%	5%	0.22
**DSG3**	Desmoglein 3	1%	5%	0.22
**DSG4**	Desmoglein 4 isoform 2	1%	5%	0.22
**DSG4**	Desmoglein 4 isoform 2	1%	4%	0.31
**EPS8**	EpidermalGrowth factor receptor pathway substrate 8	1%	5%	0.22
**ESR1**	Estrogen receptor 1	1%	6%	0.20
**FCRL3**	Fc receptor-like 3 precursor	1%	6%	0.20
**FECH**	Ferrochelatase	1%	4%	0.31
**FGFR2**	FibroblastGrowth factor receptor 2	1%	5%	0.22
**GRIA1**	Glutamate receptor, ionotropic, AMPA1	1%	4%	0.31
**GSR**	Glutathione reductase	1%	5%	0.22
**HLA-A_V2**	Major histocompatibility complex, class I, A	1%	4%	0.26
**IRF8**	Interferon regulatory factor 8	1%	5%	0.22
**ITGA2B**	IntegrIn alpha 2b preproproteIn	1%	6%	0.20
**ITGA9**	Integrin, alpha 9 precursor	1%	6%	0.20
**KIF1B**	Kinesin-like protein KIF1B (Klp)	1%	4%	0.31
**MGST1**	MicrosomalGlutathione S-transferase 1	1%	7%	0.17
**OAT**	Ornithine aminotransferase	1%	7%	0.17
**PCDH1**	Protocadherin 1 isoform 2 precursor	1%	5%	0.22
**PCDHB7**	Protocadherin beta 7 precursor	1%	2%	0.52

Suplemental Table 1. Reactivities of patient and control sera on protein micoarrays*

**PEMT**	Phosphatidylethanolamine N-methyltransferase	1%	4%	0.31
**SHMT2**	Serine hydroxymethyltransferase 2	1%	4%	0.26
**SLC38A2**	Solute carrier family 38, member 2	1%	7%	0.16
**TACR3**	Tachykinin receptor 3	1%	1%	0.78
**TGFBR1**	TransformingGrowth factor, beta receptor I	1%	1%	0.78
**TGFBR3**	TransformingGrowth factor, beta receptor III	1%	6%	0.20
**UCP3**	Mitochondrial uncoupling protein 3 (UCP 3)	1%	7%	0.17
**UPK1A**	Uroplakin 1A	1%	1%	0.78
**UPK3A**	Uroplakin 3A precursor	1%	1%	0.78
**ABCB8**	ATP-binding cassette transporter 8	1%	5%	0.15
**ACADL**	Acyl-CoA dehydrogenase, long-chain specific	1%	3%	0.26
**ACADVL-V2**	Acyl-CoA dehydrogenase, very-long-chain specific	1%	4%	0.17
**ACSL1**	Acyl-CoA synthetAse long-chAin fAmily member 1	1%	5%	0.15
**AIFM1**	Angiotensin II receptorAssociated protein	1%	5%	0.15
**ANXA11**	AnnexinA11	1%	1%	0.52
**ANXA2**	AnnexinA2 isoform 1	1%	4%	0.17
**ATP2C1-V1**	ATPase, Ca++ transporting, type 2C, membe	1%	5%	0.15
**BCKDHA**	Branched chain keto acid dehydrogenase E1, alpha polypeptide	1%	7%	0.12
**BDH1**	D-beta-hydroxybutyrate dehydrogenase	1%	7%	0.12
**CACNA1S-S1**	CalciumChannel, voltagedependent, L type,	1%	3%	0.26
**CACNA2D4**	CalciumChannel, voltagedependent, alpha	1%	6%	0.13
**CD77, A4GALT**	Slpha 1,4-galactosyltransferase	1%	7%	0.12
**CD8A**	CD8a molecule	1%	4%	0.17
**CDH15**	Cadherin 15 preproprotein	1%	4%	0.17
**CDH18**	Cadherin 18, type 2 preproprotein	1%	3%	0.26
**CHRM1**	Cholinergic receptor, muscarinic 1	1%	4%	0.17
**CHRM3**	Cholinergic receptor, muscarinic 3	1%	1%	0.52
**CHRNA1-V1**	Cholinergic receptor, nicotinic, alpha 1 isoform b	1%	5%	0.15
**CHRNA7**	Cholinergic receptor, nicotinic, alpha 7	1%	4%	0.17
**CHRNE**	Cholinergic receptor, nicotinic, epsilon	1%	2%	0.35
**COL14A1**	Collagen, type XIV, alpha 1	1%	7%	0.10
**COL18A1**	Collagen, type XVIII, alpha 1	1%	7%	0.10
**COL24A1**	Collagen, type XXIV, alpha 1	1%	5%	0.15
**COX4I2**	Cytochrome c oxidase subunit IV isoform 2	1%	1%	0.52
**COX7A2**	Cytochrome c oxidase polypeptide VIIa-liver/heart	1%	7%	0.10
**CYC1**	Cytochrome c1, heme protein	1%	3%	0.26
**DSG1**	Desmoglein 1	1%	4%	0.17
**DSG1_EC2**	Desmoglein 1	1%	4%	0.17
**DSG1_ECA**	Desmoglein 1	1%	6%	0.13
**DSG1-EC3**	Desmoglein 1	1%	5%	0.15
**DSG3_1-161**	Desmoglein 3	1%	6%	0.13
**DSG3-EC2**	Desmoglein 3	1%	5%	0.15
**ETFDH**	Electron transfer flavoprotein-ubiquinone oxidoreductase	1%	4%	0.17
**EVPL-S1**	Envoplakin, segment 1	1%	1%	0.52
**GLUD1**	Glutamate dehydrogenase 1	1%	4%	0.21
**GRIN2C**	Glutamate receptor, ionotropic, N-methyl D-aspartate 2C	1%	4%	0.21
**HLA-DPB1**	Major histocompatibility complex, class II, DP alpha 1	1%	6%	0.13
**LONP1**	lon peptidase 1, mitochondria	1%	5%	0.15
**MCCC2**	Methylcrotonyl-CoA carboxylase beta chain	1%	6%	0.13
**MUT**	Methylmalonyl-CoA mutase	1%	4%	0.17
**NDUFA3**	NADH-ubiquinone oxidoreductase B9 subunit	1%	7%	0.10
**NDUFB1**	NADH-ubiquinone oxidoreductase MNLL subunit	1%	7%	0.12
**NDUFC2**	NADH-ubiquinone oxidoreductase subunit B14.5b	1%	4%	0.17
**NDUFV3**	NADH-ubiquinone oxidoreductase 9 kDa subunit	1%	6%	0.13

Suplemental Table 1. Reactivities of patient and control sera on protein micoarrays*

**NLN**	Neurolysin, mitochondrial precursor	1%	3%	0.26
**NNT-V1**	NAD(P) transhydrogenase	1%	4%	0.17
**PCDH17**	Protocadherin 17 precursor	1%	4%	0.21
**PCDHB2**	Protocadherin beta 2 precursor	1%	4%	0.21
**PCDHB4**	Protocadherin beta 4 precursor	1%	2%	0.35
**PDGFRB**	Platelet-derivedGrowth factor receptor beta	1%	3%	0.26
**PDHA2**	Pyruvate dehydrogenase E1 component alpha subunit, testis-specificform	1%	3%	0.26
**PDK2**	Pyruvate dehydrogenase [lipoamide]] kinase isozyme 2	1%	3%	0.26
**PDK3**	Pyruvate dehydrogenase [lipoamide]] kinase isozyme 3	1%	4%	0.17
**PERP**	PerP, TP53 apoptosis effector	1%	5%	0.15
**PKP1**	Plakophilin 1 isoform 1b	1%	1%	1.05
**SDHB**	Succinate dehydrogenase (ubiquinone) iron-sulfur protein	1%	4%	0.17
**SDHC**	Succinate dehydrogenase cytochrome b560 subunit	1%	5%	0.15
**SLC25A13**	Solute carrier family 25 (aspartate/glutamate carrier), member 13	1%	4%	0.21
**SLC25A27**	Solute carrier family 25, member 27	1%	4%	0.17
**SLC7A10**	solute carrier family 7, member 10	1%	4%	0.17
**TK1**	Thymidine kinase	1%	6%	0.13
**ACADVL-V1**	Acyl-CoA dehydrogenase, very-long-chain specific	0%	4%	0.10
**ACSL4**	Acyl-CoA synthetAse long-chAin fAmily member 4	0%	5%	0.07
**ALDH2**	Aldehyde dehydrogenase	0%	2%	0.17
**ANXA10**	AnnexinA10	0%	5%	0.07
**ANXA7**	Annexin VII isoform 2	0%	1%	0.26
**ATP2C1-V4**	ATPase, Ca++ transporting, type 2C, membe	0%	7%	0.06
**CACNA1G**	VoltagedependentCalciumChannel alpha 1G	0%	4%	0.10
**CACNB4**	CalciumChannel, voltagedependent, beta 4	0%	4%	0.09
**CACNG5**	Voltagedependent calcium channelGamma5	0%	5%	0.07
**CD28**	CD28 molecule	0%	4%	0.09
**CD62P, SELP**	Selectin P (granule membrane protein 140kDa, antigen CD62)	0%	6%	0.07
**CDH6**	Cadherin 6, type 2 preproprotein	0%	4%	0.10
**CHRM2**	Cholinergic receptor, muscarinic 2	0%	5%	0.07
**CHRM5**	Cholinergic receptor, muscarinic 5	0%	3%	0.13
**CHRNA2**	Cholinergic receptor, nicotinic, alpha 2	0%	4%	0.10
**CHRNB2**	Cholinergic receptor, nicotinic, beta 2	0%	6%	0.07
**CHRNE**	Cholinergic receptor, nicotinic, epsilon	0%	7%	0.06
**COX5A**	Cytochrome c oxidase polypeptide Va	0%	6%	0.07
**COX7A1**	Cytochrome c oxidase polypeptide VIIa-heart	0%	4%	0.09
**DSC2**	Desmocollin 2 isoform Dsc2a preproprotein	0%	4%	0.09
**DSG1-EC4**	Desmoglein 1	0%	7%	0.06
**DSG3**	Desmoglein 3	0%	5%	0.07
**DSG3_S1**	Desmoglein 3	0%	7%	0.05
**EGFR**	EpidermalGrowth factor receptor	0%	4%	0.10
**EPS8L1-V2**	EPS8-like 1, isoform b	0%	3%	0.13
**ETFB-V1**	Electron transfer flavoprotein beta-subunit (Beta-ETF), isoform 2	0%	4%	0.10
**EVPL-S3**	Envoplakin, segment 2	0%	1%	0.26
**F2R**	Coagulation factor II (thrombin) receptor	0%	4%	0.10
**F2RL3**	Coagulation factor II (thrombin) receptorlike 3	0%	5%	0.07
**GRB14**	Growth factor receptor-bound protein 14	0%	6%	0.07
**GRIN2D**	Glutamate receptor, ionotropic, N-methyl D-aspartate 2D	0%	5%	0.07
**GSTK1-V1**	Glutathione S-transferase kappa 1,isoform b	0%	6%	0.07
**GSTK1-V2**	Glutathione S-transferase kappa 1,isoform a	0%	7%	0.06
**HADHB**	Trifunctional enzyme beta subunit	0%	7%	0.06
**ITGA3**	IntegrIn alpha 3 isoform a precursor	0%	5%	0.07
**ITGB3**	Integrin beta chain, beta 3 precursor	0%	6%	0.07
**ITGB3BP-V2**	Integrin beta 3 binding protein, isoform 2	0%	4%	0.09

Suplemental Table 1. Reactivities of patient and control sera on protein micoarrays*

**ITGBL1**	Integrin, beta-like 1	0%	4%	0.09
**NDUFA12**	NADH dehydrogenase [ubiquinone] 1 alpha subcomplex subunit 12;	0%	6%	0.07
**NDUFA6**	NADH-ubiquinone oxidoreductase B14 subunit	0%	5%	0.07
**NDUFB3**	NADH-ubiquinone oxidoreductase B12 subunit	0%	6%	0.07
**NDUFB7**	NADH-ubiquinone oxidoreductase B18 subunit	0%	7%	0.06
**NDUFB8**	NADH-ubiquinone oxidoreductase ASHI subunit	0%	7%	0.05
**NDUFB9**	NADH-ubiquinone oxidoreductase B22 subunit	0%	7%	0.05
**NDUFS5**	NADH-ubiquinone oxidoreductase 15 kDa subunit	0%	9%	0.04
**NME4**	Nucleoside diphosphate kinase	0%	5%	0.07
**NR3C2**	Nuclear receptor subfamily 3, group C, member 2	0%	4%	0.09
**PCDHB3**	Protocadherin beta 3 precursor	0%	4%	0.10
**PCDHB8**	Protocadherin beta 8 precursor	0%	4%	0.09
**PRDX6**	Peroxiredoxin 6	0%	7%	0.06
**SLC25A3**	solute carrier family 25 (mitochondrial carrier; citrate transporter), member 3	0%	2%	0.17
**SLC25A32**	Solute carrier family 25, member 32	0%	3%	0.13
**SLC38A7**	Solute carrier family 38, member 7	0%	6%	0.07
**UCP1**	Mitochondrial brown fat uncoupling protein 1 (UCP 1) (Thermogenin)	0%	4%	0.09
**UQCRC1**	Ubiquinol-cytochrome C reductase complex core protein I	0%	7%	0.05
**UQCRH**	Ubiquinol-cytochrome C reductase complex 11 kDa protein	0%	5%	0.07
**VDAC1**	Voltage-dependent anion-selective channel protein 1	0%	6%	0.07
**ABCB9-V4**	ATP-binding cassette, sub-family B (MDR/TAP), member 9	0%	6%	0.00
**ACSL5**	Acyl-CoA synthetAse long-chAin fAmily member 5	0%	1%	0.00
**ALAS2**	Aminolevulinate, delta-, synthase 2	0%	6%	0.00
**ALDH1B1**	Aldehyde dehydrogenase X	0%	6%	0.00
**ATP5A1**	ATP synthase alpha chain	0%	4%	0.00
**BCAT2**	Branched-chain amino acid aminotransferase	0%	4%	0.00
**BCKDK-V1**	Branched chain ketoacid dehydrogenase kinase , isoform a	0%	1%	0.00
**CACNA1S**	CalciumChannel, voltagedependent, L type,	0%	3%	0.00
**CD31, PECAM1**	Platelet/endothelial cell adhesion molecule	0%	6%	0.00
**CD54 ICAM1**	Intercellular adhesion molecule 1	0%	7%	0.00
**CD6**	CD6 molecule	0%	7%	0.00
**CD62L, SELL**	Selectin L	0%	7%	0.00
**CD87, PLAUR**	Plasminogen activator, urokinase receptor	0%	3%	0.00
**CHRNA1-V1**	Cholinergic receptor, nicotinic, alpha 1 isoform b	0%	1%	0.00
**CHRNA2**	Cholinergic receptor, nicotinic, alpha 2	0%	7%	0.00
**CHRNA9**	Cholinergic receptor, nicotinic, alpha 9	0%	6%	0.00
**CHRNE**	Cholinergic receptor, nicotinic, epsilon	0%	6%	0.00
**CHRNG**	Cholinergic receptor, nicotinic,Gamma	0%	6%	0.00
**CRHR2**	Corticotropin releasing hormone receptor 2	0%	1%	0.00
**CTNND1-V2**	Catenin, delta 1 isoform 1ABC	0%	2%	0.00
**DCI**	Enoyl-CoA delta isomerase 1	0%	1%	0.00
**DSC1**	Desmocollin 1 isoform Dsc1a preproprotein	0%	6%	0.00
**DSG1**	Desmoglein 1	0%	4%	0.00
**DSG2**	Desmoglein 2	0%	4%	0.00
**DSG4**	Desmoglein 4 isoform 2	0%	5%	0.00
**ESR2**	Estrogen receptor 2	0%	6%	0.00
**EVPL**	Envoplakin	0%	1%	0.00
**FCRL1**	Fc receptor-like 1	0%	4%	0.00
**FRS3**	FibroblastGrowth factor receptor substrate 3-	0%	4%	0.00
**FXC1**	Mitochondrial import inner membrane translocase subunit TIM9 B	0%	5%	0.00
**GLUD2**	Glutamate dehydrogenase 2	0%	5%	0.00
**GRIN2B**	Glutamate receptor, ionotropic, N-methyl D-aspartate 2B	0%	5%	0.00
**GRINA**	Glutamate receptor, ionotropic, N-methyl D-aspartate-associated protein 1 (glutamate binding)	0%	5%	0.00
**IGF1**	Insulin-like growth factor 1	0%	7%	0.00

Suplemental Table 1. Reactivities of patient and control sera on protein micoarrays*

**ITGAM**	Integrin alpha M precursor	0%	7%	0.00
**ITGB7**	Integrin, beta 7	0%	5%	0.00
**NDUFA13**	NADH dehydrogenase [ubiquinone] 1 alpha subcomplex subunit 13	0%	4%	0.00
**NDUFB6**	NADH-ubiquinone oxidoreductase B17 subunit	0%	7%	0.00
**NDUFC1**	NADH-ubiquinone oxidoreductase KFYI subunit	0%	5%	0.00
**OGDH**	Oxoglutarate (alpha-ketoglutarate) dehydrogenase (lipoamide)	0%	2%	0.00
**OTC**	Ornithine carbamoyltransferase	0%	5%	0.00
**PCDH18**	Protocadherin 18 precursor	0%	2%	0.00
**PCDHGC5**	ProtocadherinGamma subfamily C, 5 isoform 1	0%	5%	0.00
**PCK1**	Phosphoenolpyruvate carboxykinase, cytosolic [GTP]	0%	5%	0.00
**PDP2**	Pyruvate dehydrogenase [Lipoamide]]-phosphatase 2	0%	4%	0.00
**PMPCA**	Mitochondrial processing peptidase alpha subunit	0%	2%	0.00
**PRDX5**	Peroxiredoxin 5	0%	5%	0.00
**RNASEL**	Ribonuclease L (2',5'-oligoisoadenylate synthetase-dependent)	0%	4%	0.00
**RTN4**	Reticulon 4 (Neurite outgrowth inhibitor)	0%	7%	0.00
**SLC25A21**	Solute carrier family 25 (mitochondrial carrier; ornithine transporter) member 21	0%	7%	0.00
**SLC25A6**	solute carrier family 25 (mitochondrial carrier; citrate transporter), member 6	0%	8%	0.00
**TGFBR2-V1**	TransformingGrowth factor, beta receptor II	0%	6%	0.00
**TIMM10**	Mitochondrial import inner membrane translocase subunit TIM10	0%	6%	0.00
**TIMM17A**	Mitochondrial import inner membrane translocase subunit TIM17 A	0%	7%	0.00
**UQCRB-V2**	Ubiquinol-cytochrome C reductase complex 14 kDa protein, isoform 2	0%	6%	0.00

*** The letter V with a number after the gene symbol indicates the protein trascriptional variant.**
