# Supplementary material for: Pemphigus Vulgaris Autoantibody Profiling by Proteomic Technique
Source: PLoS One. 2013 Mar 7;8(3):e57587. doi: 10.1371/journal.pone.0057587 (PMC3591405; doi:10.1371/journal.pone.0057587)
Supplement: Table S2 — Reactivities of Dsg3-positive patients’ sera on protein micoarrays. (PDF) [file pone.0057587.s002.pdf]

Suplemental Table 2. Reactivities of Dsg3-positive patients' sera on protein micoarrays.*

Symbol	Number of patients	Percent of patients
DSC3-V1**	114	54%
DSC1	114	54%
HLA-DRA_V2	113	53%
PKP3	112	53%
CHRM3	109	51%
ATP2C1-V4	106	50%
CD88, C5AR1	106	50%
COL21A1_V2	106	50%
ChrnE-B	106	50%
ANXA8L1	104	49%
ITGA2	102	48%
ITGB5	101	48%
PCDH12	101	48%
CD1D	97	46%
DSG3-EC4	96	45%
CD59	96	45%
ANXA5	93	44%
COL2A1	92	43%
CD80	92	43%
CD23, FCER2	92	43%
ChrM1-a	91	43%
CD44	91	43%
ITGB3BP-V1	91	43%
HLA-DQB1	90	42%
DSC3-V2	90	42%
CD86	89	42%
HLA-G	88	42%
HLA-DQA1	87	41%
COL1A1	85	40%
HLA-DRB1	85	40%
CD26, DPP4	83	39%
HLA-DPA1	83	39%
CD49e, ITGA5	83	39%
IL1RAPL2	82	39%
PC	82	39%
PMP22	81	38%
CHRNA1-V1	81	38%
CDH1	81	38%
PCDHAC2	81	38%
CHRNB4-V1	81	38%
PERP	79	37%
CACNA1C	79	37%
CD56, NCAM2	78	37%
ANXA3	78	37%
OGFR	77	36%
FCRLA	77	36%
CHRNA5	77	36%
DSG1-EC5	77	36%
ACTA2	76	36%
PDHA1	76	36%
PMPCB	76	36%
CHRM4	75	35%
CACNG6	74	35%
ITGA4	73	34%
CHRM1	72	34%
FH	72	34%
CRAT	72	34%

Suplemental Table 2. Reactivities of Dsg3-positive patients' sera on protein micoarrays.*

MLYCD	72	34%
CD16a, FCGR3A	72	34%
PCDH10	71	33%
CD14	71	33%
ITGA7	71	33%
CD48	71	33%
CACNG2	70	33%
PCDHB12	70	33%
PANX1	70	33%
JUP-V2	69	33%
Chrn?10-B	69	33%
CD32, FCGR2B	68	32%
CD46	68	32%
CD4	68	32%
SDG1_Sc mix1	68	32%
COL8A1	68	32%
MAOB	67	32%
CHRND	67	32%
CDH4	66	31%
FDXR-V2	65	31%
PRODH	65	31%
SLC6A6-V1	65	31%
DSC2	64	30%
CHRNB1	64	30%
SOD3	64	30%
DSG1-EC1	64	30%
CD3E	63	30%
CD93	63	30%
CDH8-1	63	30%
HLA-DQB2	63	30%
CTNNA3-V2	63	30%
CD40	63	30%
HLA-E	62	29%
TGFBRAP1	62	29%
NDUFA13-3	61	29%
CHRNA1-V1-2	61	29%
FGR	61	29%
CD37	60	28%
CD66c, CEACAM6	59	28%
SOD2	59	28%
PDK4	59	28%
ALDH4A1	59	28%
DSG3-EC3	59	28%
ATP2C1-V2	58	27%
NMNAT2	57	27%
ME3	57	27%
FRS2	57	27%
FLT3	56	26%
CHRNA5	56	26%
NDUFS6	56	26%
CHRNA2-2a_SC mix	56	26%
HLA-E_V2	55	26%
CD79a	55	26%
CD63	54	25%
ETFB-V2	54	25%
PXMP4	54	25%
CDH3	54	25%
CD33	53	25%

Suplemental Table 2. Reactivities of Dsg3-positive patients' sera on protein micoarrays.*

CD42B, GP1BA	53	25%
COL25A1	53	25%
ORAI1-V2	53	25%
COL23A1	53	25%
CD36	53	25%
CTNNBL1	53	25%
CCKBR	53	25%
TIMM44	52	25%
DSG3-EC5	52	25%
ITGA8	52	25%
CHRNB4-V1	51	24%
CD64A, FCGR1A	51	24%
ANXA9	51	24%
CHRM4	51	24%
CD81	50	24%
ChrnE-mix	50	24%
CYB5B	50	24%
EPS8L2	49	23%
NDUFA9	49	23%
ChrnE-M	48	23%
FCGR2B	48	23%
AKAP10	48	23%
SYNJ2BP	48	23%
CHRNB3	48	23%
GK2	48	23%
ATP2C1-V3	48	23%
CD34	47	22%
ETFA-V1	47	22%
ABAT-V1	47	22%
NDUFV3-V2	47	22%
PGR	47	22%
PCDHB5	47	22%
DSG1	46	22%
CHRNB4-V2	46	22%
CHRNA4-V1	46	22%
MET-V1	46	22%
NDUFV3-A	46	22%
CHRNB1	46	22%
HLA-C_V2	46	22%
CHRNA9	45	21%
ITGB1	45	21%
EPS15L1	45	21%
PCDHB15	45	21%
CD69	45	21%
CPT1B	45	21%
SDG1_SC mix2	45	21%
CTNNBIP1	45	21%
CHRNB2	45	21%
DSC1	44	21%
ACTA1	44	21%
CD19	44	21%
NDUFB10	44	21%
DSG3_EC1_BL	44	21%
CD100, SEMA4D	43	20%
NR3C1	43	20%
BDKRB2	43	20%
CD75, ST6GAL1	43	20%
CD3E	42	20%

Suplemental Table 2. Reactivities of Dsg3-positive patients' sera on protein micoarrays.*

CD40_V2	42	20%
CDH9	42	20%
CD3G	42	20%
TIMM13	42	20%
DSG1_EC3_BL	42	20%
PMPCA	42	20%
CTNNAL1	42	20%
COL6A2	41	19%
ATP2A2-V1	41	19%
FGFR1-V5	41	19%
ME2	41	19%
CDH17-1	41	19%
NDUFB4	41	19%
CD98, SLC7A5	40	19%
HMGCL	40	19%
GLUD2-2	40	19%
SLC38A1	40	19%
CACNA2D1	40	19%
GRIA3	40	19%
CD74	39	18%
HADH	39	18%
TMLHE	39	18%
COL9A1_V2	39	18%
PDGFRA	39	18%
GPAM	39	18%
AR	38	18%
CTNNA1	38	18%
CD20, MS4A1	37	17%
CD98, SLC3A2	37	17%
CD14	37	17%
CD11C, ITGAX	37	17%
SLC7A8	37	17%
HBE1	36	17%
PCDHB9	36	17%
HK2	36	17%
CD66e,CEACAM5	36	17%
DSG1-EC2	36	17%
FCRLB	36	17%
MUPCDH	36	17%
SDHA	36	17%
COX17	36	17%
COL21A1	35	17%
COX10	35	17%
CD4	35	17%
HLA-A	35	17%
HLA-DPB1_V2	35	17%
NDUFA2	35	17%
CPT1A	35	17%
FECH-V1	35	17%
DSC2_SC mix	34	16%
NDUFA12	34	16%
DSG3_S2_SCBL21	34	16%
CKMT1B	34	16%
ATP2A2-V1	34	16%
CHRNA7	33	16%
CACNB4	33	16%
MTX1	33	16%
TIMM9	33	16%

Suplemental Table 2. Reactivities of Dsg3-positive patients' sera on protein micoarrays.*

BDKRB1	33	16%
OGFRL1	33	16%
CHRNA2-1a_SC mix	33	16%
ChrM1-b	32	15%
CD42a, GP9, GPIX	32	15%
SLC36A4	32	15%
CD3D	32	15%
NDUFA4	32	15%
SDHD	32	15%
ANXA9	31	15%
PIGR	31	15%
DSG2	31	15%
CD99	31	15%
CD8B	31	15%
PCDHB10	31	15%
COX5B	31	15%
NDUFS1	31	15%
ATP2A2-V2	31	15%
ABCB7	31	15%
COL9A1	30	14%
HLA-DRB1	30	14%
CD13, ANPEP	30	14%
ITGA5	30	14%
MCCC2-1	30	14%
SUOX	30	14%
PCDH8-2	30	14%
PDK1	30	14%
FCRL5	29	14%
CD207	29	14%
SLC16A10	29	14%
MAOA	29	14%
CD11A, ITGAL-2	28	13%
COL20A1_V2	28	13%
CD10, MME	28	13%
CD96	28	13%
CD1D	28	13%
CD2	28	13%
ATP2A2-V2	28	13%
DHODH	28	13%
COX11	28	13%
IGHG2	27	13%
COL3A1	27	13%
NDUFB2	27	13%
UCP2	27	13%
DSG3_EC2_BL	27	13%
GK-V1	27	13%
CD71, TFRC	26	12%
GOT1	26	12%
ITGB2	26	12%
CDH13	26	12%
SLC25A5	26	12%
EPS8L1-V1	26	12%
CD36	25	12%
HLA-B	25	12%
CACNG7	25	12%
CD18, ITGB2	25	12%
UPK3B	25	12%
DSG3-V1	25	12%

Suplemental Table 2. Reactivities of Dsg3-positive patients' sera on protein micoarrays.*

CD1B	25	12%
APLNR	25	12%
DSG3_S1_TM	25	12%
NDUFV1	25	12%
F2RL1	25	12%
CD50, ICAM3	24	11%
MTRR-V1	24	11%
COL1A2	24	11%
GRIN2A	24	11%
ITGB6	24	11%
DSG4	24	11%
FACL6	23	11%
CD92, SLC44A1	23	11%
CDS2	23	11%
CTNNA2	23	11%
GOT2	23	11%
MTRR-V2	22	10%
PKP4	22	10%
FGFRL1	22	10%
CRHR1	22	10%
CACNA1G-S2	22	10%
C1QBP	22	10%
DSG1_BL21	22	10%
CD69	21	10%
PMPCB	21	10%
CD5	21	10%
ORAI1-V1	21	10%
SLC25A17	21	10%
CD57, B3GAT1	20	9%
NDUFS6	20	9%
UCRC	20	9%
CHRND	20	9%
HTRA2	20	9%
HRH2	20	9%
CACNA1G-S3	20	9%
ECHS1	20	9%
COQ3	19	9%
CD41, ITGA2B-2	19	9%
CD66b, CEACAM8	19	9%
NGFRAP1	19	9%
CDH11	19	9%
NDUFA7	19	9%
SLC9A6	19	9%
NDUFV2	19	9%
MIPEP	18	8%
CYCS	18	8%
CD52	18	8%
EPS8L3	18	8%
DSG3_EC5_BL	18	8%
UQCRQ	17	8%
CACNA1S-s3	17	8%
DSC3_S1_TM	17	8%
PPIF	17	8%
IGF1R	17	8%
BAD	17	8%
CHRNA2-1b_SC mix	17	8%
FLT3LG	17	8%
ACTG2	16	8%

Suplemental Table 2. Reactivities of Dsg3-positive patients' sera on protein micoarrays.*

DSG2	16	8%
MTX2-V1	16	8%
NDUFV3-V1	16	8%
Chrn?10-B1	16	8%
NDUFA1	16	8%
CD29, ITGB2	16	8%
FCGRT	16	8%
AGTR1	16	8%
DSG1	15	7%
SCP3	15	7%
FPGS	15	7%
TIMM22	15	7%
CD3G	14	7%
UQCRFS1	14	7%
CD9	14	7%
TSPO	14	7%
CKMT2	14	7%
DSC3	14	7%
PTH1R	13	6%
CD8A	13	6%
CD47	13	6%
CD97	13	6%
CD66d,CEACAM3	13	6%
SLC25A19	13	6%
SLC25A15	13	6%
CD58	13	6%
CHRM2	13	6%
SLC36A2	13	6%
COLQ	13	6%
HSPD1	13	6%
CDH24	13	6%
CDH16	13	6%
CD27	12	6%
ANXA4	12	6%
CD1A	12	6%
CD28	12	6%
CD55	12	6%
NDUFV1-V1	12	6%
NDUFA13-2	12	6%
SLC25A39	12	6%
TACR1	12	6%
COL5A1	12	6%
CD66f, PSG1	12	6%
PCDH20	12	6%
HLA-DQA1	12	6%
SLC25A20	12	6%
GCDH-V1	11	5%
WARS2	11	5%
ITGB1BP1	11	5%
CD19	11	5%
IGHG2	11	5%
CD90, THY1	11	5%
AKAP1	11	5%
SCP2	11	5%
CD89, FCAR	11	5%
COX6A1	11	5%
OXA1L	11	5%
NDUFB1-b	11	5%

Suplemental Table 2. Reactivities of Dsg3-positive patients' sera on protein micoarrays.*

UQCRC2	10	5%
FCRL4	10	5%
ANXA8L2	10	5%
PXMP3	10	5%
BCL2	10	5%
CD40	10	5%
FLT4	10	5%
CD83	10	5%
PMP2	10	5%
TOMM40	10	5%
ITGAV	10	5%
CD94, KLRD1	10	5%
PCCB	10	5%
CDH7	10	5%
CHRNA9	10	5%
CD86	10	5%
NDUFS4	10	5%
Chrn?10-mix	10	5%
NDUFB5	10	5%
VDAC2	10	5%
CD79b	9	4%
CS	9	4%
HLA-F	9	4%
CD73, NT5E	9	4%
UQCRB	9	4%
BCKDK-V2	9	4%
Chrn?10-mix	9	4%
IGFBP2	9	4%
SLC25A14	9	4%
IGF2R	9	4%
CD24	9	4%
PCDHB1	9	4%
CHRNA6	9	4%
DSC1	9	4%
HSPE1	9	4%
ACTC1	9	4%
ATP5O	9	4%
HLA-C	9	4%
GPX4	9	4%
LARS2	9	4%
PCCA	9	4%
DSG1_EC4_BL	9	4%
MTX2-V2	9	4%
ITGB1BP2	8	4%
MDH2	8	4%
ANXA1	8	4%
IGF2	8	4%
TOMM70A	8	4%
TOMM20	8	4%
AK2	8	4%
DSG1	8	4%
CHRNB3	8	4%
COX4I1	8	4%
CD5	8	4%
CD68	8	4%
SLC25A12	8	4%
MCCC1	8	4%
HLA-DQB1	8	4%

Suplemental Table 2. Reactivities of Dsg3-positive patients' sera on protein micoarrays.*

FCRL2	7	3%
CACNA1G-S2	7	3%
HLCS	7	3%
DLAT	7	3%
DSG4_SC mix2	7	3%
PCDHB11	7	3%
CD80	7	3%
ITGAX	7	3%
CD43, SPN	7	3%
ANXA6	7	3%
COL4A3BP	7	3%
PCDHB13	7	3%
MYCBP	7	3%
TIMM8B	7	3%
COX6A2	7	3%
CD85, LILRB1	7	3%
CACNA1S-s2	7	3%
DSG3_25-88_BL	7	3%
UPK2	6	3%
CDH20	6	3%
PERP	6	3%
PERP	6	3%
GRB7	6	3%
PDGFRL	6	3%
GPT	6	3%
SLC25A17	6	3%
ChrM1-b1	6	3%
NDUFS3	6	3%
COL9A3	6	3%
PDHX-V1	6	3%
TIMM17B	6	3%
DSG3	6	3%
CD95, FAS	6	3%
CHRM5	6	3%
CHRFAM7A	6	3%
CD70	6	3%
CDH2	6	3%
CTNNB1	6	3%
CHRNA3	6	3%
CACNG3	6	3%
PCDH21	6	3%
CD1C	6	3%
HRH1	6	3%
NNT-1	6	3%
ITGAL-1	6	3%
GATM	6	3%
DIABLO	6	3%
OXCT1	6	3%
DSG2	6	3%
CPT2	5	2%
CD7	5	2%
COL4A6	5	2%
ACTB	5	2%
SLC25A1	5	2%
ATP2C1-V2	5	2%
HLA-DRA	5	2%
TK2	5	2%
SLC6A6-V3	5	2%

Suplemental Table 2. Reactivities of Dsg3-positive patients' sera on protein micoarrays.*

COL19A1	5	2%
GRB2	5	2%
CACNG4	5	2%
SHMT1	5	2%
PERP	5	2%
CHRNA6	5	2%
CDH19	5	2%
TIMM23	5	2%
AGTRAP	5	2%
PPOX	5	2%
NDUFS2	5	2%
ITGB8	5	2%
CHRNA1-V1-3	5	2%
AK3	5	2%
CTNND1-V1	5	2%
NDUFA5	5	2%
SURF1	5	2%
CDH23	5	2%
CD83	5	2%
CHRNA4-V2	5	2%
ALAS1-V1	5	2%
DSG3_S2_TM	5	2%
BCAT2	5	2%
UCRC	5	2%
CD9	5	2%
COX6C	5	2%
FDX1	5	2%
CACNB1	5	2%
HRH4	5	2%
SLC25A11	5	2%
NFS2	5	2%
NFS1	5	2%
SLC25A4	5	2%
COL10A1	5	2%
PERP	5	2%
DSG4	5	2%
CTNNA3-V1	4	2%
CKMT2	4	2%
DSC2	4	2%
BCKDHB-V1	4	2%
PCDHB14	4	2%
DBT	4	2%
F2RL2	4	2%
SLC25A10	4	2%
HCCS	4	2%
DNAJA3-V1	4	2%
SLC25A31	4	2%
ACTG1	4	2%
C5orf39	4	2%
DLD	4	2%
GRIA2-V2	4	2%
PERP	4	2%
CACNA1G-S1	4	2%
ATP5A1	4	2%
PCDHB16	4	2%
COL20A1	4	2%
HK1-V4	4	2%
NDUFA10	4	2%

Suplemental Table 2. Reactivities of Dsg3-positive patients' sera on protein micoarrays.*

PKP2	4	2%
ABCD4	4	2%
DSG3-EC1	4	2%
ACTN2	4	2%
UPK1B	4	2%
CDH12	4	2%
ACADSB	4	2%
JUP-V1	4	2%
DSG1_EC1_BL	4	2%
DSG3_EC4_BL	4	2%
HADHA	4	2%
SLC7A13	4	2%
VDAC3	4	2%
TOMM40	4	2%
AK3L1-V7	4	2%
SLC25A2	4	2%
ABCB6	4	2%
CHRNB4-V2	3	1%
TST	3	1%
CD72	3	1%
ACADS	3	1%
CD70	3	1%
COL6A1	3	1%
ACSL3	3	1%
MLYCD	3	1%
ATP2C1-V1	3	1%
YWHAE	3	1%
SLC25A18	3	1%
HLA-A_V2	3	1%
ATP2C1-V3	3	1%
CD84	3	1%
CD82	3	1%
CD38	3	1%
CD8B	3	1%
CD66a, CEACAM1	3	1%
GSR	3	1%
CHRNA7	3	1%
ATP7B	3	1%
ESR1-V4	3	1%
KIF1B	3	1%
DSG4_SC mix1	3	1%
DSG1_1-496_BL	3	1%
UCP3	3	1%
CD1A	3	1%
FCRL3	3	1%
MGST1	3	1%
DSG3-V2	3	1%
SHMT2	3	1%
SLC38A2	3	1%
FECH	3	1%
ITGA2B-1	3	1%
DSG3_EC3_BL	3	1%
EPS8	3	1%
PEMT	3	1%
CHRNA3	3	1%
DSG3 #20_SC mi	3	1%
CACNG1	2	1%
CKMT1A	2	1%

Suplemental Table 2. Reactivities of Dsg3-positive patients' sera on protein micoarrays.*

IRF8	2	1%
PCDH1	2	1%
CTNND2	2	1%
PDGFRB	2	1%
DSG4	2	1%
ITGA9	2	1%
BDH1-V2	2	1%
LONP1	2	1%
GRIA1	2	1%
DSC3_S2_TM	2	1%
TGFBR3	2	1%
OAT	2	1%
PCDHB7	2	1%
SLC7A10	2	1%
CD77, A4GALT	2	1%
HLA-DPB1	2	1%
ETFDH	2	1%
CHRNA1-V1	2	1%
DSG1-EC3	2	1%
DSG1	2	1%
SLC25A13	2	1%
CD8A	2	1%
TK1	2	1%
CDH15	2	1%
CHRNA7	2	1%
ANXA2	2	1%
ATP2C1-V1	2	1%
EVPL-S1	2	1%
ACADVL-2	2	1%
ANXA11	2	1%
PCDH17-2	2	1%
NLN-1	2	1%
DSG3_1-161_BL	2	1%
CACNA1S-s1	2	1%
GRIN2C	2	1%
MUT	2	1%
CACNA2D4	2	1%
SDHC	2	1%
SLC25A27	2	1%
DSG3-EC2	2	1%
NDUFC2	2	1%
PDK2	2	1%
CDH18	2	1%
ChrnE-M1	2	1%
MCCC2-2	2	1%
GLUD1	2	1%
PDHA2	2	1%
NDUFV3-B	2	1%
NDUFB1-a	2	1%
NDUFA3	2	1%
DSG1_ECA_BL	2	1%
DSG1_EC2_BL	2	1%
PDK3	2	1%
BCKDHA	2	1%
COX7A2	2	1%
SDHB	2	1%
NNT-2	2	1%
COL24A1	2	1%

Suplemental Table 2. Reactivities of Dsg3-positive patients' sera on protein micoarrays.*

ACADL	2	1%
COX4I2	2	1%
CYC1	2	1%
COL14A1	2	1%
ITGB1BP3	1	0%
TGFBR1	1	0%
FGFR2	1	0%
CKMT1A	1	0%
ACADM-V3	1	0%
ACSL1	1	0%
COL18A1	1	0%
PERP	1	0%
AIFM1	1	0%
CD28	1	0%
ABCB8	1	0%
PCDHB2	1	0%
ChrM1-a1	1	0%
DSG3	1	0%
EPS8L1-V2	1	0%
ALDH2	1	0%
DSC2	1	0%
ACADVL-1	1	0%
ChrnE-mix	1	0%
CHRM5	1	0%
ITGBL1	1	0%
DSG1-EC4	1	0%
NME4	1	0%
NDUFA6	1	0%
CHRNA2	1	0%
CD62P, SELP	1	0%
PRDX6	1	0%
NDUFB8	1	0%
CHRNB2	1	0%
CDH6	1	0%
F2RL3	1	0%
NDUFB7	1	0%
CACNG5	1	0%
GRIN2D	1	0%
COX7A1	1	0%
GSTK1-V1	1	0%
EVPL-S3	1	0%
UQCRH	1	0%
ANXA10	1	0%
COX5A	1	0%
PCDHB3	1	0%
NDUFB9	1	0%
F2R	1	0%
ITGB3BP-V2	1	0%
ACSL4	1	0%
CACNB4	1	0%
PCDHB8	1	0%
GRB14	1	0%
ATP2C1-V4	1	0%
NR3C2	1	0%
SLC25A3	1	0%
NDUFB3	1	0%
UQCRC1	1	0%
SLC38A7	1	0%

Suplemental Table 2. Reactivities of Dsg3-positive patients' sera on protein micoarrays.*

CHRM2	1	0%
NDUFS5	1	0%
UCP1	1	0%
DSG3_S1_SCBL21	1	0%
EGFR	1	0%
CACNA1G	1	0%
HADHB	1	0%
ETFB-V1	1	0%
GSTK1-V2	1	0%
CHRFAM7A	0	0%
CHRNG	0	0%
ANXA13	0	0%
UPK3A	0	0%
TACR3	0	0%
UPK1A	0	0%
CHRNA4-V2	0	0%
CHRNA4-V1	0	0%
CCKAR	0	0%
PCDHB4	0	0%
ITGB3	0	0%
PKP1	0	0%
CHRM3	0	0%
ALDH1B1	0	0%
NDUFA12	0	0%
ANXA7	0	0%
ITGA3	0	0%
VDAC1	0	0%
SLC25A32	0	0%
CD6	0	0%
CTNND1-V2	0	0%
CD54 ICAM1	0	0%
CD87, PLAUR	0	0%
CD31, PECAM1	0	0%
DSC1	0	0%
ATP5A1	0	0%
NDUFB6	0	0%
PCDHGC5	0	0%
NDUFA13-1	0	0%
RNASEL	0	0%
DSG1	0	0%
CHRNG	0	0%
CHRNA1-V1	0	0%
CHRNA9	0	0%
CD62L, SELL	0	0%
RTN4	0	0%
BCAT2	0	0%
BCKDK-V1	0	0%
EVPL	0	0%
NDUFC1	0	0%
CACNA1S	0	0%
CHRNA2-2b_SC mix	0	0%
PMPCA	0	0%
PCK1	0	0%
ITGB7	0	0%
UQCRB	0	0%
PDP2	0	0%
ABCB9-V4	0	0%
TIMM17A	0	0%

Suplemental Table 2. Reactivities of Dsg3-positive patients' sera on protein micoarrays.*

IGF1	0	0%
ALAS2-V2	0	0%
GRINA	0	0%
TGFBR2-V1	0	0%
FCRL1	0	0%
FXC1	0	0%
PRDX5	0	0%
OTC	0	0%
FRS3	0	0%
ITGAM	0	0%
ESR2-Vc	0	0%
GRIN2B	0	0%
TIMM10	0	0%
ChrnE-B1	0	0%
DCI	0	0%
DSG4	0	0%
DSG2	0	0%
SLC25A6	0	0%
SLC25A21	0	0%
GLUD2-1	0	0%
OGDH-V1	0	0%

*The total number of patients' sera was 212, because it included Dsg3 antibody positive samples identified by standard ELISA.
** Letter V with a number after the gene symbol indicates the protein trascriptional variant.
